# Deep Learning Approaches for Automated Prediction of Treatment Response in Non-Small-Cell Lung Cancer Patients Based on CT and PET Imaging

**DOI:** 10.3390/tomography11070078

**Published:** 2025-06-30

**Authors:** Randy Guzmán Gómez, Guadalupe Lopez Lopez, Victor M. Alvarado, Froylan Lopez Lopez, Eréndira Esqueda Cisneros, Hazel López Moreno

**Affiliations:** 1 TecNM/Centro Nacional de Investigación y Desarrollo Tecnológico (TecNM/CENIDET), Interior Internado Palmira s/n, Cuernavaca 62493, Morelos, Mexico; d20ce076@cenidet.tecnm.mx (R.G.G.); victor.am@cenidet.tecnm.mx (V.M.A.); 2 San Peregrino Cancer Center, Republica de Ecuador 103 (int 407), Las Americas, Aguascalientes 20230, Aguascalientes, Mexico; lopezoncodr@gmail.com (F.L.L.); dra.ere.esqueda.cp@gmail.com (E.E.C.); hazellopez246@gmail.com (H.L.M.)

**Keywords:** deep learning, lung cancer treatment response, CT imaging, PET imaging, morphology analysis, metabolic analysis

## Abstract

The rapid growth of artificial intelligence, particularly in the field of deep learning, has opened up new advances in analyzing and processing large and complex datasets. Prospects and emerging trends in this area engage the development of methods, techniques, and algorithms to build autonomous systems that perform tasks with minimal human action. In medical practice, radiological imaging technologies systematically boost progress in the clinical monitoring of cancer through the information that can be analyzed in these images. This review gives insight into deep learning-based approaches that strengthen the assessment of the response to the treatment of non-small-cell lung cancer. This systematic survey delves into the various approaches to morphological and metabolic changes observed in computerized tomography (CT) and positron emission tomography (PET) imaging. We highlight the challenges and opportunities for feasible integration of deep learning computer-based tools in evaluating treatments in lung cancer patients, after which CT and PET-based strategies are contrasted. The investigated deep learning methods are organized and described as instruments for classification, clustering, and prediction, which can contribute to the design of automated and objective assessment of lung tumor responses to treatments.

## 1. Introduction

Cancer is the leading cause of mortality worldwide. In particular, lung carcinoma is one of the most frequent tumors and causes the highest number of deaths [1]. According to statistics from the International Agency for Research on Cancer [2], the cancers with the highest incidence in descending order were breast, lung, colorectal, prostate, skin, and gastric cancer. Notably, lung cancer accounts for a significant portion of cancer-related deaths. In 2020, 22% of the 8,164,372 cancer deaths globally were attributed to lung cancer. In comparison, 11.45% were due to colorectal cancer, 10.17% to liver cancer, 9.42% to gastric cancer, and 8.39% to breast cancer. Among various types of cancer, non-small-cell lung cancer (NSCLC) is one of the leading causes of death worldwide for both men and women.

Over the decades, the public health sector has developed advanced and practical treatments for cancer. Drug and radiation treatments for cancer include chemotherapy and radiotherapy. Surgery is also a common treatment option. However, the overall survival rate of lung cancer patients is at most five years in some cases [3]. In light of this, monitoring methods have also been devised to assess the efficacy of these treatments. However, there are significant challenges in treatment progress and monitoring mechanisms. The valuation of treatments needs to be enhanced in accuracy and speed to know the tumor response and perform in due course. It will, in turn, prevent toxicity and wasted time dispensing treatments that are not adequate for patients. Currently, there are two standard ways to monitor therapeutic responses. The first method finds morphological changes and follows anatomical details through computed tomography (CT), which scans the internal structure of the body. The second way is to discern metabolic changes by positron emission tomography (PET) [4]. It is a nuclear medicine technique that builds a three-dimensional image to show functional information about the cancer patient. Both methods aid in assessing the response of the tumor to the treatment and guide further actions.

**Figure 1:**
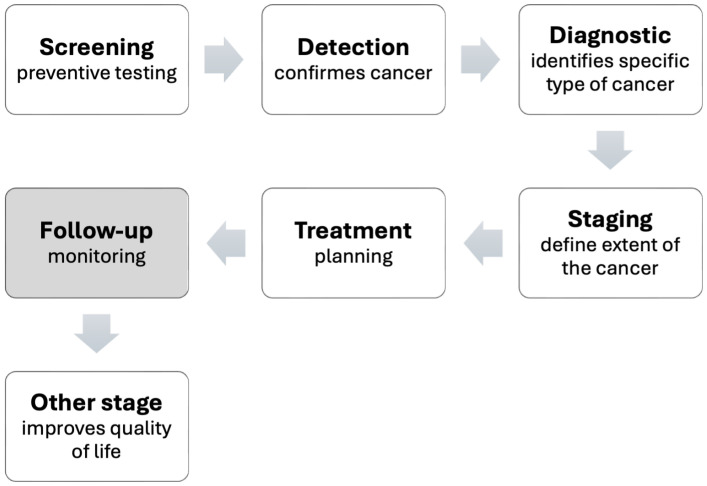
Stages of cancer care.

Artificial intelligence (AI) has emerged as a transformative force in various fields and has boosted changes in medical practice. We use the general term AI to designate the field in which the technology and advances that concern us in the health sector are developed. The integration of machine learning (ML) and deep learning (DL), two branches of AI, into radiology methods is a trend. This fusion paves the way for more skilled extraction of quantitative data from images and promises a future for more effective disease diagnosis and treatment. We use the term DL to designate specific developments that use deep neural networks (DNN) to process medical images. As a matter of fact, medical imaging and computational tools are combined in a term frequently encountered in the literature as radiomics [5]. This field uses AI tools to extract intrinsic knowledge from medical images or data. It is a method that enlightens specialists about the intensity, shape, size, volume, or structure of tissues. For example, gauging these variables is vital in treating and monitoring cancer. Thus, the radiomics approach offers a practical solution to the critical challenge of cancer cure. In clinical practice, the workflow for cancer care can be broken down into several key steps summarized in Figure 1. The current literature review investigates AI-based approaches for assessing patient responses to lung cancer treatments, which are indicated in Figure 1 under the “Follow-up” block.

Clinical evaluation of anti-cancer drugs and treatments through imaging poses a challenge for specialists. Due to the expertise required and the time needed to analyze a large number of medical images, this task is often burdensome. The opportunities presented by AI technologies are worth taking into account in these types of applications. The goal of this literature survey is to examine research that sheds light on developments via DL outcomes in measuring how a lung cancer patient responds to treatment. The screened DL-based methods primarily rely on CT or PET images.

The survey not only addresses advancements in AI technology but also explores practical issues related to integrating intelligent systems into the healthcare sector. In this regard, we examine the regulations and legislation governing AI systems in health services, along with the validation protocols essential to introduce safe and efficacious systems and devices in healthcare facilities. Furthermore, the survey discusses the main challenges associated with the transition to AI technology within public healthcare systems.

This paper is structured as follows: Section 2 and Section 3 provide insights into imaging techniques used to assess lung cancer and establish standardized clinical criteria for monitoring patients’ response to treatments through CT and PET scans. Section 4 explains DL models and associated elements that make medical image processing feasible and defines the key metrics for evaluating the performance of deep neural networks (DNN). Section 5 gets to the heart of the matter and focuses on literature research about DL methods to assess lung cancer treatments. Section 6 introduces the existing regulations and legislation governing the deployment of AI technology in the medical field and explains how these initiatives are evolving. It also addresses validation protocols for AI technologies based on medical images. Finally, challenges, future implications, and knowledge gaps for integrating these advancements into cancer treatment workflows are discussed. Section 7 gathers significant topics from related surveys and explores the current research in the field. Section 8 recalls critical issues and provides a closing discussion. Finally, the paper concludes with a summary of our findings.

## 2. Imaging Studies for Lung Cancer

The main histological types of lung cancer include non-small-cell lung cancer (NSCLC), small-cell lung carcinoma (SCLC), and neuroendocrine tumors (NET). Their marking is essential in treating this disease, as it is worth noting that NSCLC accounts for 85–90% of all lung cancers. NSCLC can be further categorized into squamous-cell carcinoma (SCC), adenocarcinoma (AC), and large-cell carcinoma (LCC). It is also noticeable that SCC and AC are responsible for about 80% of all lung cancers [6].

In the field of oncology, imaging plays a vital role in cancer screening, diagnosis, treatment monitoring, and prognosis. These practices rely on details from clinical histories and various types of physical exams. Preliminary examinations can include blood tests, pulmonary function tests, and cytological evaluations. Blood tests (BTs) measure or examine substances in the blood. Some of the routine BTs for lung cancer are the complete blood count (CBC) and the liquid biopsies studies [7]. Pulmonary function tests (PFTs) measure lung volume, capacity, flow rates, and gas exchange. PFTs for lung cancer control include spirometry, lung volume, total lung capacity, and diffusion capacity tests [8,9,10]. Cytological tests examine cells and can utilize samples from broncho-alveolar lavage, bronchial washings, bronchial brush smears, pleural fluid, or sputum [7]. Additionally, effective disease management requires histopathological studies interpreted by pathologists and other imaging studies assessed by radiologist. The latter interpret the medical images to determine the pathological type and the malignancy level of cancer.

Standard anatomic imaging techniques for staging and restaging in patients with NSCLC include chest X-ray, ultrasound, computed tomography (CT), Magnetic Resonance Imaging (MRI), and studies using nuclear medicine. Moreover, technologies are playing a role in nuclear medicine. Positron Emission Tomography (PET) has evolved to become a routine test to control solid tumors, including lung cancer. Also, with nuclear medicine technology, there are specialized studies. For example, somatostatin receptor scintigraphy (SRS) is helpful if lung neuroendocrine tumors are present [6]. The biomarkers of structural and functional images may coincide in the details they provide, but some data are distinctive to each method. PET can detect functional abnormalities before they can be perceived by established structural imaging. On the other hand, histopathological images involve microscopic images of tissues or cells removed from the patient by a biopsy or surgery. This imaging modality is considered the gold standard for cancer detection. However, it is an invasive technique, and, as with other modalities of medical images, their interpretation may be subjective.

With the development of deep-learning-based medical tools, clinicians can make more accurate and quicker detections, measurements, or classifications of lung nodules or tumors. Medical imaging assisted with deep learning is an emerging research area with applications in different imaging techniques. Wang, 2022 [11] discussed possible research directions and challenges involved in integrating deep learning and various medical imaging modalities for varied clinical objectives. Related investigations include studies of lung cancer centered on MRI scans, such as the works by Moon et al. (2023) and Fan et al. (2021) [12,13]. The first study analyzed the survival of NSCLC after surgery. The second work dealt with metastasis diagnosis. Other standard analyses are conducted via chest X-rays with deep learning techniques. We cite the work reported by Shimazaki et al. (2022) and Lu et al. (2020) [14,15], which addressed the problem of lung cancer detection. Histopathological images also play a role in the deep learning approach. Rajasekar et al. (2023); Liu (2023); Li and Thapa (2021); and Hatuwal et al. (2020), for instance, developed deep learning methods for lung cancer diagnosis based on histopathological images [16,17,18,19].

It is well established that CT has been the foremost standard imaging method for NSCLC staging. CT scans can lead to an accurate measure of the tumor size. On the other hand, PET is feasible and more accurate for assessing treatment response. In this context, functional imaging and mixed techniques have a growing role in the NSCLC control. A combined PET/CT scan has been introduced to produce more precise images. Deep learning approaches for lung cancer detection have also been built on combined PET/CT scans. Applications include differentiating benign nodules and solid lung cancer and metastasis prediction Zhong et al. (2023) [20].

The screening of the various clinical imaging modalities and related literature presented above provides insight into the scope of deep learning and the breadth of engagements of these techniques in cancer detection and evaluation. From now on, this survey is restricted to CT and PET imaging. It is guided toward reviewing strategies for a more automatic and accurate evaluation of the response to treating patients with lung cancer.

### 2.1. Computed Tomography

Computed tomography ( CT) is derived from X-ray imaging and has been improved by computer technology. The obtained scans are three-dimensional images of any anatomical region. The function of a CT scan is to measure the transmission of X-rays through the patient in multiple projections. The projections are produced by the X-ray tube rotating around the patient. The machine’s computer processes the received signals to generate cross-sectional images or “slices”. Successive slices can be “stacked” digitally to form a three-dimensional image of the patient that allows the basic internal structures to be identified [21].

### 2.2. Positron Emission Tomography

Positron emission tomography is an imaging technique in nuclear medicine similar to a CT scan. A key difference is that PET needs radiopharmaceuticals to be injected into the patient’s bloodstream. As an outcome, images of internal biochemical processes are acquired. This technique allows for measuring regional glucose consumption and quantifying metabolic activity [22].

## 3. Lung Cancer Treatment Evaluation Methods

**Figure 2:**
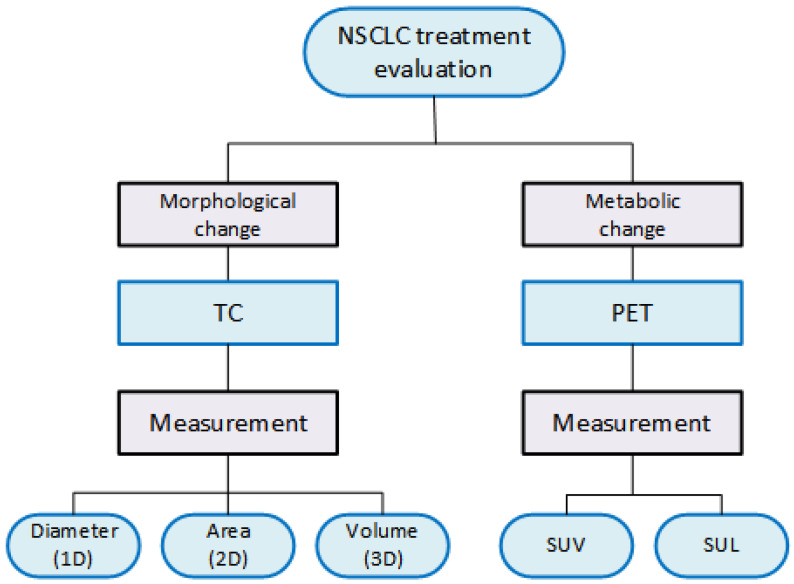
Types of measurements used in the evaluation of NSCLC treatments.

Medical scanners deliver a series of cross-sectional images of internal organs in patients. These images are two-dimensional and constructed in slices. However, the slice fusion allows for a three-dimensional (3D) visualization of the organs. Before interpretation, it is essential to assess the 2D or 3D dataset from the scans to identify any abnormalities or signs of cancer in the patient. Following image acquisition, the first step in this process is to select the appropriate slice interval(s). On that account, the radiologist frames the study region. Depending on the scanning technology used, the decision-making process can vary. Our review deals with two standard approaches that lay the groundwork for tumor evaluation, which are broadly outlined in Figure 2.

When monitoring treatment for NSCLC, CT images expose morphologic changes in the patient’s lesions. Instead, PET images reveal metabolic changes. Lung lesion tests guided by these means are performed before and after treatment to measure the lesion. Clinical trials have used various criteria to assess the efficacy of cancer treatment. However, there have been successful efforts to standardize this evaluation process in medical practice. The viable measures acquired from CT images include the diameter, area, or volume of the lesions. Conversely, in PET images, the key measure pertains to the absorption volume of a radiopharmaceutical. More precisely, the quantitative indicators to characterize the lesion in PET scans are the Standardized Uptake Value (SUV) and the Standard Uptake Lean Body Mass (Figure 2).

The standard clinical criteria for evaluating the response to treatment in NSCLC patients based on CT and PET scans are discussed below, together with advances in this domain. This research highlights that accurately assessing the response to cancer treatment remains a significant challenge. Notably, AI and DL have gradually made their way into the medical field, offering promising solutions and potential for future advancements in cancer treatment evaluation.

**Figure 3:**
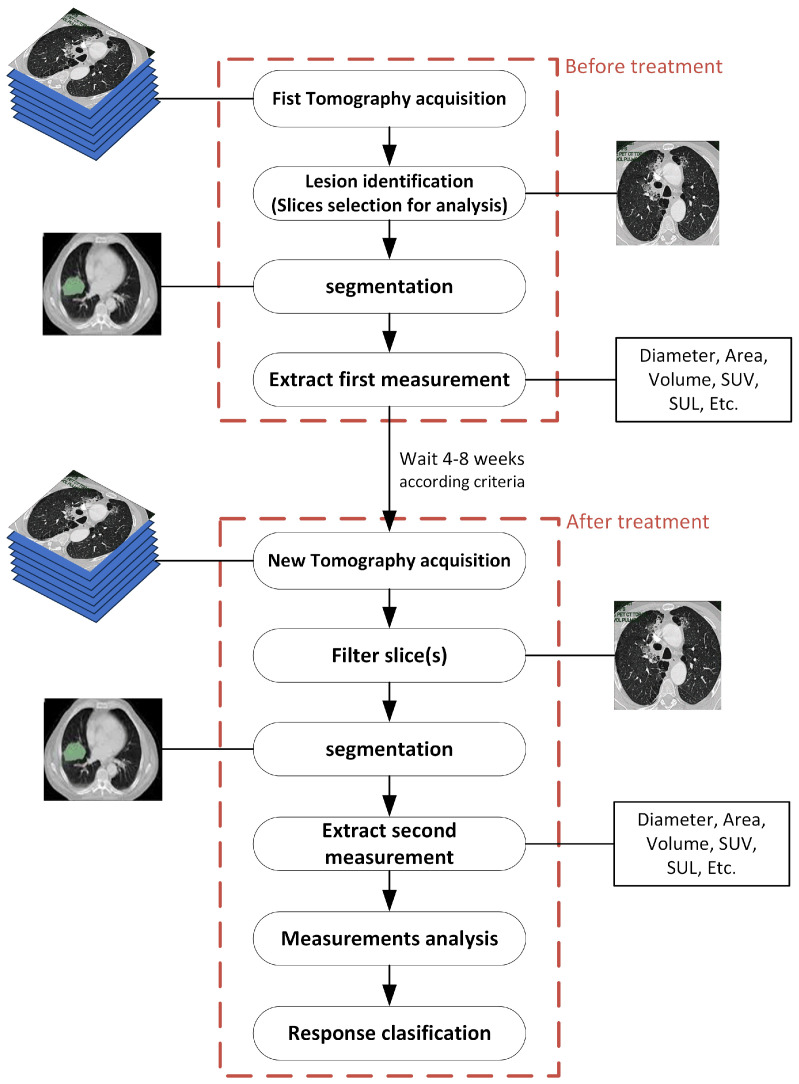
General methodology for evaluating treatment assisted by deep learning.

With the aim to enable CT and PET image processing with DL numerical tools, It is crucial to emphasize features such as resolution, contrast, and noise to ensure measurement. A comprehensive flowchart for cancer treatment evaluation, aided by deep learning, is shown in Figure 3. The sequence made before the treatment is repeated a few weeks later with the same level of detail and for the same slice intervals. DL-based tools allow for a thorough examination of tumor burden.

### 3.1. Criteria to Assess Morphological Changes

In 1981, the World Health Organization (WHO) published guidelines that established standard criteria for assessing the effectiveness of cancer treatments in clinical trials. This initial method focused on determining the significance of anticancer treatments by estimating tumor area size. In 2000, a group for developing the Response Evaluation Criteria in Solid Tumors (RECIST) submitted new guidelines for the oncology community. Their emphasis shifted to measuring morphological changes to monitor cancer treatments though CT scans. This approach is based on the observation that the simple sum of the maximum diameters of individual tumors better reflects cell destruction than the two-dimensional product used by the WHO method. Since the launch of RECIST 1.1 in 2009, it has become the preferred criterion to assess treatment response in cancer clinical trials and to aid in developing new treatments for this disease. However, the field is not without its challenges. New criteria based on earlier versions of RECIST have emerged in the literature. Summing up, these appearing approaches have expanded their application to specific types of treatments and addressed the monitoring of particular cancers. Current research also explores the pros and cons of both area and volume measurements obtained from CT images to determine which measurements correlate more effectively with patient survival. So far, it has not been conclusively demonstrated that area and volume measurements evaluate better the treatment response than unidirectional measurement in the target lesion. Table 1 compiles comprehensive literature relevant to this research topic from oncology and radiology researches.

The sequence of steps followed in a clinical evaluation of cancer with images culminates in measuring lesions. The primary objective of monitoring cancer treatment is to compare imaging scans taken before and after treatment. This process concludes in applying the RECIST guidelines to determine the effectiveness of the treatment or therapy. Global imaging processing begins with the choice of the “target lesion”, which serve as the primary focus during disease follow-ups. Target lesions should be chosen based on their variability, the difficulty of measurement, and after the specifying of the cut-off interval in the CT images [29]. RECIST defines four primary criteria for assessing target lesions. Additionally, there are three possible responses for non-target lesions (see Table 2). It is worth noting that the success in evaluating a partial response confirms the efficacy of assessing the overall response to cancer treatment [30].

Evaluations conducted in 2D or 3D relying on criteria for 1D measurements can result in inaccuracies. The four-response classification defined in RECIST 1.1 may not reflect changes in the area or volume of the target lesion after cancer treatment. Over time, guidelines have been revised to improve the assessment of area and volume changes, as weighed in the mRECIST guidelines.

### 3.2. Criteria Based on Metabolic Changes

Functional PET images allow for predicting the spatial distribution of the metabolic or biochemical activities in patients, which is vital for diagnosing particular tumors. During PET scanning for cancer screening, glucose-mimicking tracers such as Fluorodeoxyglucose (FDG) are administered intravenously to the patient. PET images capture the varying levels of FDG concentration in tumors, quantified by the Standard Uptake Level (SUV). As tumor progression and destruction correlate with this level, SUV serves as an excellent index for measuring tumor activity. Currently, the response to cancer treatment can be surveyed in PET images via two quite different criteria. The European Organization for Research and Treatment of Cancer (EORTC) developed in 1999 the first set of criteria. The second is the PET Response Criteria in Solid Tumors (PERCIST), introduced in 2009 [31]. EORTC criteria aid in evaluating specific lesion regions of interest (ROI) chosen at the outset and monitored in subsequent scans. In this method, the SUV is adjusted for body surface area. Then, for each PET scan, the SUV is calculated as a function of the body mass (SUL) for an ROI with a maximum diameter of 12 mm. On the other hand, PERCIST is generally regarded as a more straightforward method to apply. It gives more detailed guidance on how to define target lesions [32]. The metabolic responses of the tumors according to both criteria are shown in the Table 3.

By way of alternative, designing DL methods for cancer monitoring uses the RECIST criteria to create a robust framework for both the development and evaluation of DL methods used in image analysis for oncology. These standard guidelines from medical practice can be applied in the design of DL tools to label and classify images to be used for training. Moreover, the RECIST criteria are valuable for estimating the accuracy and effectiveness of DL models. This valuation allows the models to demonstrate their ability to replicate the judgment made by specialists regarding treatment responses. The following section explains the DL models and components relevant to the design of DL devices, or more generally, assistive AL devices for healthcare.

## 4. Deep Learning Networks (DLNs)

Artificial intelligence (AI) combines hardware, software, and mathematics within computer systems to process information by emulating human actions or functions. AI uses logical reasoning to solve simple problems and to develop expert decision-making systems. Furthermore, AI empowers systems handling large volumes of data to learn and solve problems. Machine learning (ML) is a branch of AI that allows computers to learn from data. Within this framework, Deep learning (DL) is a form of ML that uses algorithms to automate tasks. Notably, DL resources are planned and built on deep neural networks, where input data is analyzed at different layers [11].

DL-based image segmentation and processing are revolutionizing the analysis of medical images and data, facilitating the assessment of how cancer tumors respond to treatment. A neural network is characterized by the neuron model, the topology of the connections between neurons, and the learning algorithm it employs. The main neural networks developed for DL applications are categorized into three classes. The first one involves unsupervised or generative feature learning. This class aims to identify a high-order correlation in observed data for pattern analysis. The second focuses on supervised learning. This class is aimed at discrimination for pattern classification. The third class comprises hybrid deep networks. These networks strive to achieve assisted discrimination by optimizing or utilizing criteria from discriminative supervised learning [11].

Several neural networks have emerged, each designed for specific purposes in image processing, and they have become valuable assets in numerous fields, especially within the healthcare sector [33]. Table 4 summarizes polular architectures for Deep Learning Neural Networks (DLNNs), along with a brief description of their applications.

### 4.1. Convolutional Neural Network

**Figure 4:**
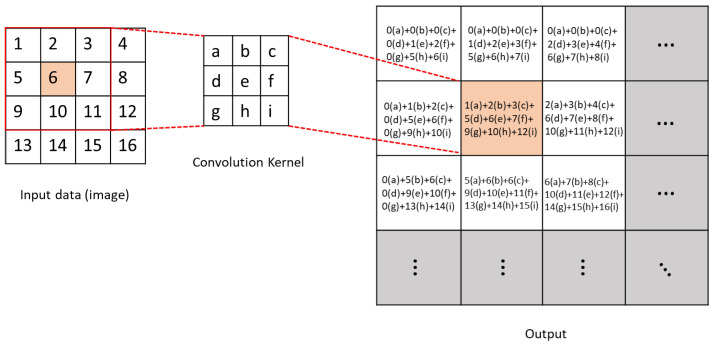
Schematic diagram of convolution operation.

DL architectures use neural networks that are built by overlaying layers. These structures, a subset of ML techniques, learn relationships and patterns from data to aid the decision-making process. One of the most widely used DL architectures is the Convolutional Neural Network (CNN). This model, introduced by LeCun et al. in 1989 [34], has achieved remarkable success in the field of computer vision. The CNN model consists of three primary layers: The convolutional layer performs a mathematical operation that calculates the value of an output pixel as a weighted sum of the neighboring pixels. This convolution operation is performed between the image and a matrix known as the convolution kernel [42]. It is show in Figure 4.

**Figure 5:**
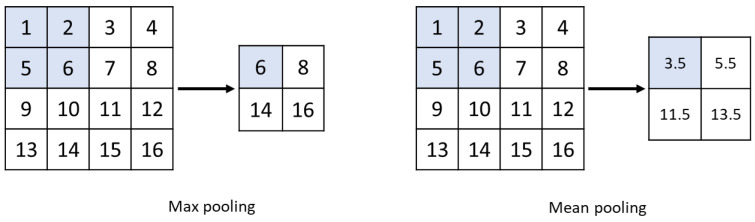
Max-pooling and mean-pooling schematic diagram.

**Figure 6:**
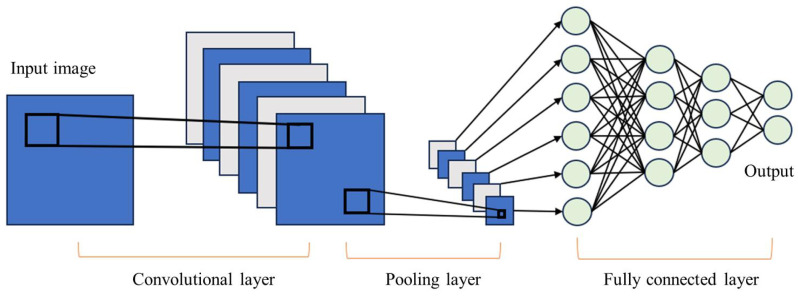
General CNN architecture.

The pooling layer or clustering layer reduces the image matrix by preserving pixels that contain the most information through techniques like max pooling or by averaging the neighboring pixels with mean pooling, as shown in Figure 5. Finally, the fully connected layer performs the classification task [43]. CNNs excel in feature extraction and have firmly established their dominance in image recognition, classification, and video recognition. Figure 6 depicts an example of a CNN structure used for image classification.

#### U-Net

**Figure 7:**
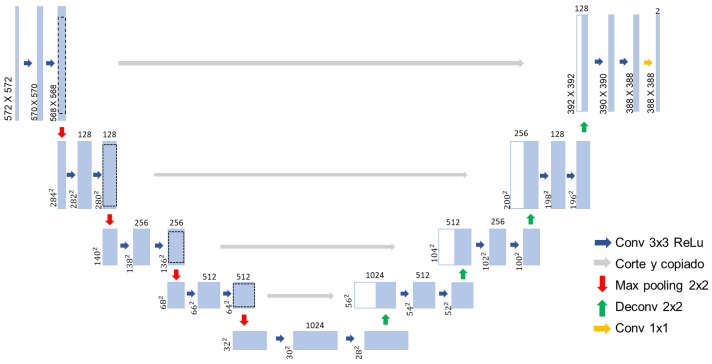
CNN U-Net architecture.

A widely adopted CNN architecture for performing segmentation tasks is U-Net which was first introduced in [44]. This architecture was specifically designed to tackle the challenge of limited annotated data in the medical field. The U-Net architecture consists of two main paths: the Contraction path and an Expansion path [45]. The Contraction path comprises convolution and pooling layers that capture contextual information and downsample the input. In contrast, the Expansion path contains deconvolution layers that decode the encoded data, utilizing information from the Contraction path via skip connections to generate a segmentation map as illustrated in Figure 7.

### 4.2. Recurrent Neural Network

**Figure 8:**
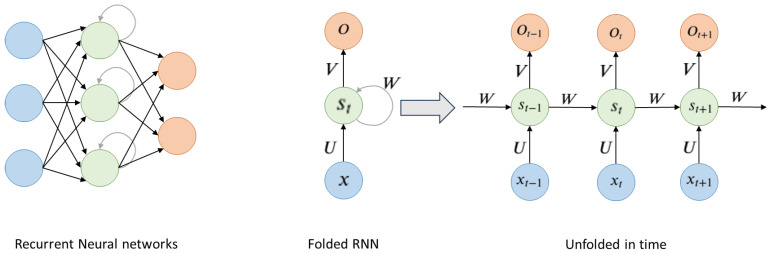
General RNN architecture.

Similarly, Recurrent Neural Networks (RNNs) are extensively applied in DL architectures. These networks are particularly suited for processing sequential data such as speech recognition and language processing. RNNs learn features of time series data by the memory of previous inputs in the internal state of the neural network. This means that past information is implicitly stored in the hidden layer, and the output for the current input is computed by considering all the previous inputs through these state vectors as shown in Figure 8. Moreover, RNN can predict future information based on past and present data [46].

As shown in Figure 8, $x_{t}$ is the input of the sequence in time *t*. $s_{t}$ is the memory unit of the sequence at time *t* and caches previous information. which is calculated by (1).

(1) $$s_{t}=\tanh(Ux_{t}+Ws_{t-1})$$

$O_{t}$ is the output of the hidden layer of the sequence at time *t*. After passing through multiple hidden layers, one can obtain the final output of the sequence at time *t* [47].

(2) $$O_{t}=\tanh(Vs_{t})$$

### 4.3. Recursive Neural Network

Another class of DL network is the Recursive Neural Network (RvNN). This architecture makes predictions in a hierarchical structures and classifies results as compositional vectors. The development of RvNN was inspired by the recursive auto-associative memory (RAAM), an architecture designed for processing objects structured in arbitrary forms, such as trees or graphs [48].

### 4.4. Deep Generative Networks

A Deep Generative Network (DGN) is a powerful tool designed for learning any data distribution through unsupervised learning. All generative models aim to accurately capture the distribution of the training dataset, thus allowing the generation of new data with certain variations. Two of the most widely used architectures for data generation in deep learning are Variational Autoencoder and Generative Adversarial Networks [49].

#### 4.4.1. Variational AutoEncoder

**Figure 9:**
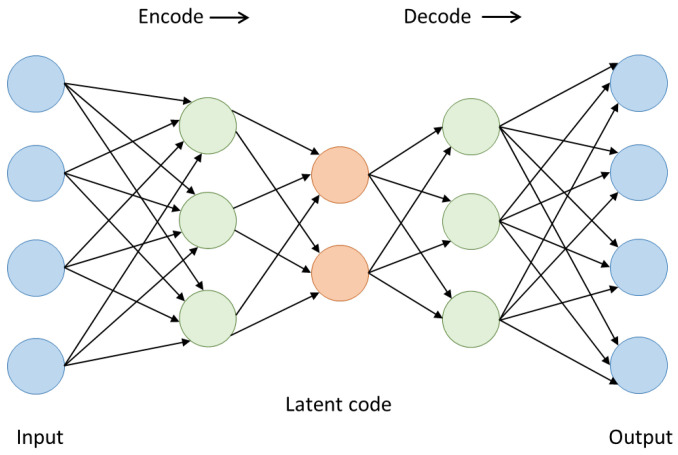
VAE architecture.

The Variational AutoEncoder (VAE) is a sort of deep generative model where simultaneous learning occurs from the data using a decoder and an encoder [50]. It is an encoder–decoder architecture. The encoder transforms the input data into a latent representation while the decoder attempts to reconstruct the original data from this representation. The encoder generates a compact, low-dimensional representation known as a “latent code”. This encoder can be built using various neural networks, like fully connected or convolutional networks. Likewise, the decoder, which aims to reconstruct the original data from the latent code produced by the encoder, can also be designed using different types of neural networks. These generative models strive to capture the underlying probability distribution of a dataset and generate new samples. The objective of the VAE is to minimize the difference between the original and reconstructed data [51]. Figure 9 illustrates the general structure of a VAE.

#### 4.4.2. Generative Adversarial Network

**Figure 10:**
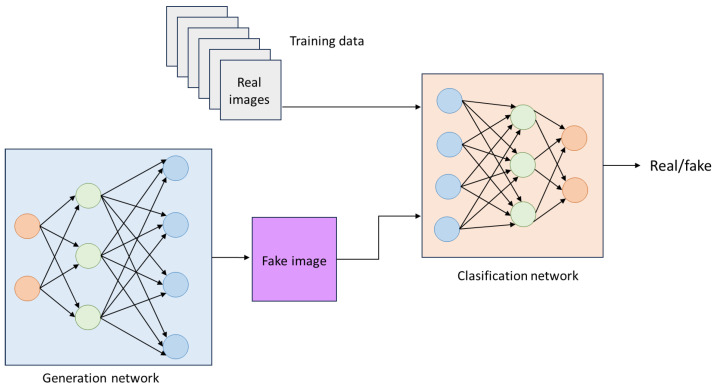
General GAN architecture.

Generative Adversarial Networks (GANs) are systems designed for data generation systems that use probabilistic models to identify patterns and structures within datasets, created by [39]. These networks consist of two main components: a generator and a discriminator. The generator produces data that closely resembles real data, while the discriminator is responsible for classifying this data to distinguish between generated and authentic data. As the generator improves its results to resemble real data more closely, the discriminator becomes increasingly adept at identifying differences between the two types of data. GANs are trained and optimized using deep neural networks. Their main objective is to enable one specific dataset to adopt the distribution characteristics of of another dataset [52]. Figure 10 shows the general structure of a GAN.

### 4.5. Activation Functions for Neural Networks

In neural network (NN) training, the activation function introduces the non-linearities that make the models learn complex functions [53]. Since the inception of neural networks, different versions of these functions have been proposed and probed. A remarkable property i of activation functions is that they must be differentiable elements. The cause is that NNs learn based on a backpropagation algorithm, which improves the predictive capabilities of NNs and facilitates updates via differentiation. However, the need for differentiability feature causes some drawbacks to be solved. In this context, the different activation functions have unique characteristics, making them more or less suitable for the different applications of neural networks. Below are the most commonly used activation functions.

**Figure 11:**
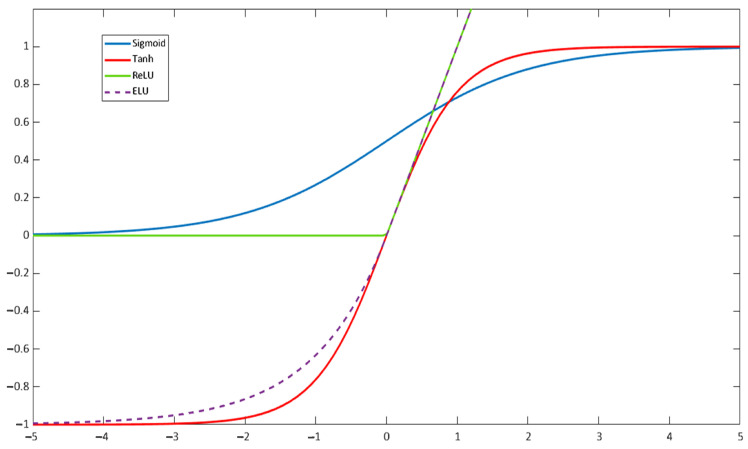
Plot of the Sigmoid, hyperbolic tangent, ReLU, and ELU functions.

The Sigmoid activation function maps the input range from (−∞;+∞) to the range in (0;1). Due to this output range, the sigmoid function tends to saturate at 0 or 1, meaning that the output of each unit is also squashed. This squashing effect leads the gradient close to zero, resulting in a vanishing gradient problem. This behavior makes it challenging to optimize after a certain point [54]. The Sigmoid function is detailed in Table 5 and its output landscape is shown in Figure 11.

The Hyperbolic Tangent function has a structure similar to that of the Sigmoid function, bit it squashes input values to the range of (−1,+1). One key difference is that the derivative of the hyperbolic tangent is steeper than that of the sigmoid, which can lead to faster convergence during training. However, like the Sigmoid function, the bounded output values of the Hyperbolic Tangent (Figure 11) can also result in vanishing gradients [55]. This function is defined in Table 5.

The ReLU (Rectified Linear Unit) function is the most commonly used activation function in Deep Learning research for its simplicity, which translates into lower computational effort [56]. The ReLU function is given in Table 5 and produces outputs ranging from 0 to infinity. It allows for the activation of hidden layers in neural networks by an output with a true zero value. However, a major downside of ReLU is that it transforms all negative values into zero. This limitation is a specific instance of the vanishing gradient problem. Thus, once the neuron gets negative, it is unlikely for it to recover [57]. The output landscape of the ReLU function is shown in Figure 11.

The Exponential Linear Unit (ELU) function was initially proposed as an improvement over ReLU in [58], showing superior performance for classification than traditional ReLU. For x≥0, ELU follows the same rule as ReLU, while for x<0, it increases exponentially, as shown in Figure 11. The main improvement of ELU over ReLU is the ability to output negative values. The ELU activation function is defined in Table 5.

### 4.6. Evaluation Metrics to Assess Deep Learning Models Performance in Image Processing

In image processing, DNN models guide tasks such as object detection, image segmentation, and classification. These models operate effectively by following two essential steps: model training and performance evaluation based on various metrics. This section provides an overview of the metrics used to rate the performance of DNN models.

Object detection models are designed to identify and locate objects within an image by bounding boxes around them and classifying each object. Segmentation is a critical step in imaging processing, regardless of the clinical application, whether it be screening, detection, diagnosis, or treatment assessment. This process divides an image into segments based on specific characteristics. It streamlines image analysis by partitioning the image into meaningful parts and filtering out irrelevant data. Image segmentation is performed using complex neural networks that analyze visual data at the pixel level. Each pixel in an image is assigned a label so that pixels with similar characteristics share the same label. These methods support advanced pattern recognition capabilities. Muller et al. (2022) [59] highlight the strong predictive capabilities of segmentation algorithms, noting that their performance often aligns closely with that of clinicians. However, they argue that the score of the model performance is frequently implemented inadequately.

The evaluation of segmentation focuses on both classification accuracy and localization correctness. Its main objective is to measure the similarity between the predicted outcome and the ground truth. In the context of image segmentation, a Region of Interest (ROI) refers to a specific area within an image that is chosen for analysis or further processing. This area typically contains the object or feature of interest, such as an organ or a lesion. The type of ROI can significantly influence the complexity of the segmentation process and the final evaluation score. For organ segmentation, the ROI is usually consistently positioned, making it relatively straightforward to assess the accuracy of the selection. In contrast, a lesion ROI can vary greatly in terms of spatial and morphological features, which may result in metrics that provide a less accurate assessment.

Then, image classification refers to determining the category of an image from a set of classes established in advance. Metrics are vital for fine-tuning and optimizing DNN-based models to enhance their performance. By examining how changes to the model affect these metrics, designers can refine their models to achieve better outcomes, focusing on the specific characteristics they aim to enhance [60].

Various metrics have been utilized in research on segmentation and classification approaches for medical images. Muller et al. (2022) [59] identified the following as the most commonly used: Dice Similarity Coefficient (DSC), Intersection-over-Union (IoU), Sensitivity (Sens) and Specificity (Spec), Accuracy/Rand Index (Acc), Receiver Operating Characteristic (ROC), and the area under the ROC curve (AUC), Cohen’s Kappa (Kap), and Average Hausdorf Distance (AHD). They also define the equations needed to compute each of these metrics. In this review, we emphasize the most commonly encountered metrics in the literature reported here for treatment assessment. Model performance metrics are intended to score the similarity between the predicted (or automatic) segmentation and the manual (or ground truth) [61] segmentation. The way to compare these metrics relies on the computation of a confusion matrix for a binary segmentation task, as demonstrated in Table 6. The elements of the matrix represent different types of predictions along with their corresponding occurrence counts: True Positives (TP), False Positives (FP), False Negatives (FN), and True Negatives (TN) [59].

The Specificity (SPE) calculates the number of actual negatives that a model correctly identifies. It evaluates the ability of a model to detect all negative instances.

(3) $$SPE=\frac{TP}{TP+FP}$$

The Sensitivity (SEN) computes the number of actual positives correctly detected. It assesses the ability of a model to identify all positive instances.

(4) $$SEN=\frac{TP}{TP+FN}$$

The Accuracy Score (ACC) measures how often the model makes correct predictions. It is calculated by adding the number of correct True Positive to the number of True Negative predictions, and then dividing this sum by the total number of predictions. This metric provides a clear indication of the performance of the model in making accurate predictions.

(5) $$ACC=\frac{TP+TN}{TP+TN+FN+FP}$$

The Dice Coefficient (DC) quantifies the similarity between the regions segmented by automatic methods and those segmented manually. It is defined in Equation (6). Essentially, this metric is used to evaluate the accuracy of the segmentation results produced by the model.

(6) $$DC=2\times \frac{\left|R_{p}\cap R_{T}\right|}{\left|A_{p}\right|+\left|A_{T}\right|}$$

where $R_{p}$ means the automatic segmentation region and $R_{T}$ is the region of ground truth. $A_{p}$ is the number of automatically segmentation region pixels and $A_{T}$ is the number of the ground truth pixels [61].

### 4.7. Image Preprocessing Techniques

#### Filters

Filtering is a preprocessing technique that modifies or improves an image. For example, a filter can amplify or attenuate certain features within the image. Filtering uses the so-called neighborhood operations, where the value of a given pixel in a region of the processed image is computed by an algorithm that considers the values of the pixels in the neighborhood. This image handling involves the average value of the surrounding pixels. This processing is akin to convolution, an algorithm that adjust the value of pixels by using information from adjacent pixels [42].

**Figure 12:**
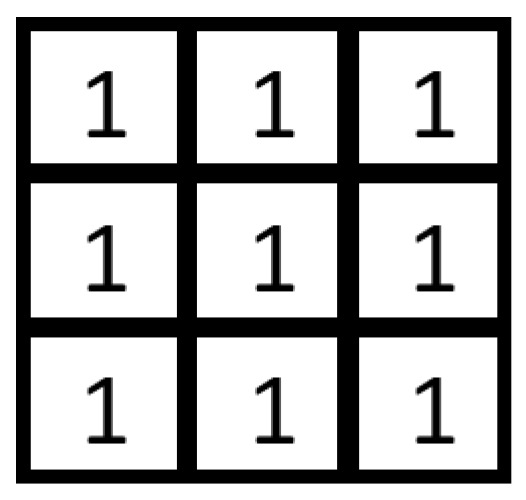
Low-pass filter mask.

The low-pass filter plays a crucial role in removing noise by decreasing the gain of high-frequency components [62]. The simplest form of low-pass filter has unity coefficients in all its elements, as depicted in Figure 12.

**Figure 13:**
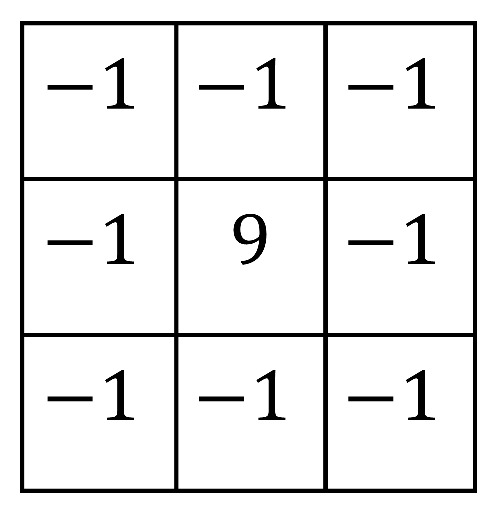
Edge highlight filter mask.

In contrast, edge enhancement increases the gain of high-frequency components, which emphasizes pixels with gray values that differ from those of their neighboring pixels. However, if the image is noisy, the algorithm will also amplify the noise. So, it is advisable to remove the noise first before applying this technique. The most commonly used mask to enhance edges is shown in Figure 13 [42].

**Figure 14:**
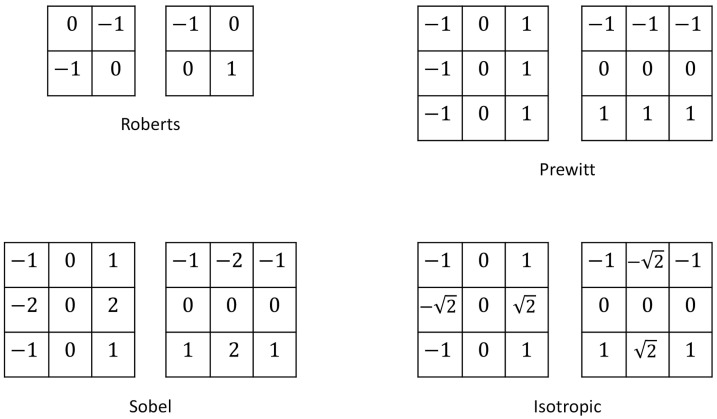
Roberts, Prewitt, Sobel, and Isotropic filter masks.

Gradient-based filters use the concept of approximated derivatives in discrete spaces and the differences between neighboring pixels. Depending on the ratios of the pixel values, these differences can give rise to one-dimensional or two-dimensional derivatives. Some of the most widely used gradient-based filters are called Roberts, Prewitt, Sobel, and Isotropic [63] filters. The masks for these filters are presented in Figure 14.

Table 7 presents some filtering options reported to be used for image preprocessing in DL methods reviewed in the literature cited in Section 5.

## 5. The Potential of Deep Learning in NSCLC Treatment Evaluation

Research-oriented efforts on DL-inspired healthcare systems are devised to aid the clinical procedures lung cancer patients must undergo. Most of the current advances in this field deal with disease diagnosis rendered by classification models built with CNNs. However, the DL field has played a role in undertaking further medical procedures. One reason for expanding the scope of application of DL during the follow-up procedures for lung cancer patients is that their prognosis is often adverse because, many times, the disease is detected late. One measure that could alleviate this prognosis would be to recognize effective treatments for each patient. Cancer treatments comprise surgery, radiation, drugs, and other therapies to cure cancer, shrink tumors, or stop the progression of cancer. The present survey delves into DL techniques that can be a part of new intelligent systems conceived to aid the monitoring of NSCLC treatments or clinical trials rooted in CT and PET images. It is crucial to note that a comprehensive survey on the subject is needed to acknowledge the full potential of these methods.

### 5.1. Underscoring the Role of Deep Learning in Measuring Morphological Changes

Morphological changes observed in cancer lesions are the standard measure to assess cancer treatments. The total number of images of a lung CT scan can vary depending on the size of the lung region being examined and the specific setup of the CT equipment. Generally, it renders hundreds of individual images to provide a detailed view of the lungs and surrounding structures. Deciding which images to evaluate and which lesions to measure can be challenging, especially when lesions have irregular shapes or are located in hard-to-access areas. As a result, different radiologists may evaluate the same lesion differently. In this context, the criteria for determining tumor evolution after treatment were established to streamline the evaluation process for specialists while preserving predictive efficiency. As Section 3.1 mentions, the RECIST guidelines provide a standardized framework that allow specialists to acknowledge changes in tumor burden and track the progression of a cancer disease. It applies only to solid tumors and facilitates assessing the images set by restricting the measurement of lesions to one-dimensional sizing of up to five target lesions. The approach is centered on measuring the largest diameter of the tumor lesions in CT scan images. It is used if at least one tumor can be measured on a CT scan. In that event, the response to treatment may fall into one of the following options: complete response (CR), partial response (PR), advancing disease (AD), and stable disease (SD). However, exploiting novel technologies can guide improved medical practice. In particular, DL makes possible the efficient processing of a large number of images using advanced algorithms. In addition, DL tools can assess other characteristics of cancer lesions, such as surface area per slice or volume of the whole lesion. These tools allow for either the rapid direct classification or the measurement of the maximum length, surface, or volume of cancer lesions, increasing the accuracy of the valuation while reducing variability. Disease monitoring aided with DL methods often uses the RECIST to train the algorithms or models built on deep neural network architectures. Figure 3 in Section 3 outlines the main stages of a DL method for cancer treatment monitoring. However, a significant challenge remains in fully automating this process, as DL methods often require manual inputs. Some research literature presented here addresses automated or semiautomated notation, segmentation, and response classification. We also discuss the efficiency of processing time later in this section.

The following studies are described to acknowledge the engaged DL tools, such as sequences of objects, algorithms, image processing, and expert decision-making systems to treat CT images. These investigations delve into the various tasks involved in DL method designs that are practical for NSCLC detection, with a focus on treatment monitoring of this disease.

The study conducted by Chang et al. [69] is first outlined to illustrate a DL approach that has been proven for analyzing CT images and assessing treatment responses in lung cancer patients. The study explains how developers can create these algorithms and highlights several key processes involved in the design of DL-based systems. The DL software developed in the cited work classified treatment responses directly by comparing CT images taken before and after chemotherapy, without isolating lesions (without segmentation). The images were characterized to differentiate between “response” and “non-response” to chemotherapy. One important step discussed in this approach is labeling, which can employ a range of ML and AI techniques. Specifically, DL-based methods can be used to identify anatomical structures and anomalies in medical images. The study provides insights into the role of deep neural networks, which are instrumental in feature extraction. This process allows the DL algorithm to recognize and select the most relevant features from the images for further analysis and processing. The deep neural networks used underwent a training phase that enabled them to classify responses to cancer treatments based on the identified features. Ultimately, the referenced work underlines the use of tools that improve computational efficiency. Overall, the cited research has proven valuable in tackling the issue of insufficient labeled data.

Focusing on the technical details, Chang et al. [69] developed a DL-based method to predict the treatment response of patients with NSCLC undergoing chemotherapy. The method uses CT images as input. A deep neural network is trained to classify the tumor response to chemotherapy. The output is the prognostic between *response* (which refers to CR and PR) and *non-response* (for PD and SD). The AI-designed cancer treatment evaluation method leverages customary DL tools for image processing which are informed by datasets from two hospitals. The backbone network was pretrained on images from the ImageNet database. The efficacy of the DL method in predicting tumor evolution was gauged by comparing its output with the decision made by the radiologists following the RECIST guidelines. The proposed approach holds three features: It is grounded on the Multiple Instance Learning (MIL) model. The MIL problem, as described further below, is a type of supervised learning where multiple observed instances are assigned to a single class label [70]. Various pretrained backbone CNNs were tested as feature-extracting networks, processing input data into a specific feature representation. The method was completed by designing an attention mechanism pooling process. This last was shaped to handle the computational resources of the model. In computer vision applications, pooling reduces the spatial dimensions of an image while essential features are kept.

The approach described involves labeling a series of images instead of each slice. In standard image classification problems, standard supervised learning trains predictive models by mapping input instances or feature vectors to outputs or labels. In such cases, the training datasets consists of input-output pairs, with each instance labeled with a specific class. An alternative scenario occurs when multiple instances share a general class statement. For example, in lung cancer images, the labels of *benign* or *alignant* can describe the overall image, or a Region Of Interest (ROI) may only be crudely defined. The so-called Multiple Instance Learning (MIL) approach is scheduled for that event. This learning method caters to weakly annotated data, where class labels are assigned globally to images or bags. The system then learns to detect relevant patterns within these images locally. MIL overcomes the constraints of standard supervised learning when individual instance labels are unavailable. A series of research works explaining the deep MIL approach are given in [71,72,73]. Ilse et al. [72] reported different methods for tackling the task of bag classification.

Many investigations deal with practical issues to improve neural network performance or test the DL tools. In this context, a specific analysis by Holliday and Dudek [74] evaluated the performance of pretrained CNNs as feature extractors in visual matching applications. The study covered various CNN architectures from different families, such as AlexNets, VGG Nets, ResNets, and DenseNets. It is to be noted that all of these CNN families were tried by Chang et al. [69], the work explained above. The CNN analysis provides insights into the robustness of CNN features under appearance, scale, and perspective variations. These findings on CNNs’ robustness can be extended to different architectures to assist in the choice of specific architectures for various applications.

Some methods, in contrast, rely on labeled images as their input. This approach reduces the level of automation because it requires manual labeling. The input data typically comes from one-dimensional measurements found in specific CT scan databasesor is provided by radiologists, who measure lesions according to RECIST guidelines. The work referenced below describes a CNN architecture that is well-suited for processing text entries derived from these prelabeled images.

Standard RECIST criteria are based on 1D measurements of lesions to evaluate therapeutic responses in solid tumors. In this context, Arbour et al. [75] presented a method for developing a DL model to estimate the best overall survival and the non-evolving disease survival after specific lung cancer treatment. This project aimed to promote the assessment of large clinical databases from a significant number of patients treated outside of clinical trials. After that, the aim was to delineate a general approach for treatment assessment. The idea was to use data retrieved from medical records rather than directly from scans. In any case, the text data was acquired from scans. The DL model was constructed with a deep network architecture to estimate the RECIST response using, as input, text from clinical radiology reports of patients with advanced NSCLC treated with PD-1/PD-L1 blockade. The deep neural network was built with encoding, interaction, and two fully connected layers with a hyperbolic tangent activation function. An output layer with a softmax activation function followed this structure. Gold-standard RECIST reports from qualified radiologists were used to train the model. The predictions made by the model showed a high degree of similarity to the RECIST categorization. A key finding was the confidence established in the standardized RECIST criteria for oncological treatment assessment. It results from the assertion that the performance of the DL-based method was consistent irrespective of the reporting style of the radiologists from the followed institutions. However, further research is necessary to establish the applicability and generalization of the method to other treatment regimens.

Still framed on the knowledge of morphological changes, a systematic path toward more intelligent monitoring systems entails, in addition to measurement in scanned images, other critical tasks, like lesion detection and segmentation. Detection involves recognizing and forecasting bounding boxes around objects and classifying them. It is a step towards segmentation, which focuses on partitioning an image into distinct regions to boost targeted analysis of specific areas. Then, the obtained data is guided to comparison and evaluation functions. Several AI techniques for pretreatment and filtering may enhance image processing and the various steps for developing support DL systems. The critical steps in image handling are segmentation and creating markers on preselected images with CNNs. The set of DL tools aims to streamline the CT image measurement procedure for radiologists. Some developments allow for an increasing degree of automation. Automated or self-configuring image processing can help mitigate inconsistent and biased measurements among radiologists. In what follows, the referenced works deal mainly with detection and segmentation. In addition to training the DL model based on RECIST outcomes for large groups of images, some approaches endeavor to translate the 1D measurement procedure dictated by these guidelines into a DL method with more or less intelligent actions. For instance, Tang et al. [76] developed a semiautomatic 1D method using a cascaded CNN to label the RECIST procedure. This method begins with a radiologist manually drawing a marked box to define the region of interest (ROI), which limits the selected area. A CNN was constructed with two cascaded deep neural networks. The first is a Spatial Transformer Network (STN) with three components: a localization network, a grid generator, and a sampler. This STN predicts translation, rotation, and scaling transformations of the lesion based on a transformation matrix. It was made to control the normalization of the lesion region, which makes the overall method robust against variability in lesion size, location, and orientation across different images. The Stacked Hourglass Network SHN was used in the cascade for RECIST estimation. After image transformation, the SHN estimates the positions of the endpoints to approximate the longest and shortest diameters of the lesion. The SHN structure involved convolutional, max pooling, and upsampling layers that enhance the accuracy of RECIST predictions.

In the same line, Xie et al. [77] discussed the challenges and various approaches to lesion detection. This research focused on developing an annotation method for images of lung cancer lesions. Their method, RECIST-Net, a CNN, was designed to detect four extreme points and the central point of the lesion.

Lesions segmentation and their succeeding conversion to unidirectional measurement per the RECIST guide are critical steps in the automation of image processing. Challenges arise when visual markers for the endpoints and surrounding areas of a lesion are hard to discern. It is also challenging when meaningful clinical interpretation or elucidation of the lesion is needed. In contrast, lesion segmentation and measurement conversion become easier to automate if the lesion boundaries are well-defined. Woo et al. [78] addressed this issues and contributed a semiautomatic method for 1D measurements in CT images using CNNs. Their approach involved three cascaded CNNs trained to label whether the size of a target lesion is larger or smaller than 32 pixels. However, the DL CNN set struggled to classify when the lesion size matched 32 pixels. The initial steps for image pretreatment were as follows: CT images were resized, and 1D measurements were converted from centimeters to pixels. The images were then enlarged by bicubic interpolation. The target lesions were placed in a 128 × 128-pixel frame with the central measurement point as the center of the frame. The method uses an arbitrary point within the target lesion as input. As a result, it is not a fully automated algorithm. However, the proposed method showed excellent agreement with measurements made by a radiologist. This work serves as a foundation for further integration of models to detect lesions, identify an arbitrary point within them, and perform measurements using the entry point to automate the process.

So far, the discussion has been oriented to aspects that impact the level of automation of DL methods and to introduce potential AI and DL techniques for CT image processing, as well as the challenges of their implementation. As mentioned before, RECIST guidelines are delineated to potentially decrease the work of radiologists due to the large number of images to process. Actually, 2D and 3D may enhance the precision of monitoring changes in lesions over time. Numerous studies have been conducted to evaluate the significance of 2D and 3D measurements. The reader is referred to the literature cited in Table 8 and Table 9. However, hereafter, we detail some research addressing volumetric segmentation of lung cancer lesions in CT images, grounded on DL methods. We also examine the time spent on image processing using DL methods and clinical assessments by specialists. Jiang et al. [79] worked with a multiscale CNN approach to volumetrically segment NSCLC tumors and nodules in patients undergoing immunotherapy, which alters the size and appearance of the tumors. This work addressed the variability of semiautomatic segmentation encountered in several methods reported in the literature. In the same vein of CT image segmentation, Chen et al. [80] developed a novel encoder–decoder-based CNN architecture to segment tumor lesions accurately.

A method that delved further into the automation of cancer lesion measurement was proposed by Kidd et al. [81]. It moved towards the volume measurement of malignant pleural mesothelioma (MPM), a rare and severe form of cancer that originates in the membrane lining the lungs and the inside of the ribs. The highlight of this strategy is its completly automated character, which allows for the evaluation of the chemotherapy response to MPM without requiring manual input. Manual annotations of specialists were only used as a means to train the segmentation model and validate MPM volumetry. This method used a CNN with a two-dimensional architecture to segment each axial slice interval of the CT. It classified the response and evaluated the performance of the DL-based method according to mRECIST criteria (a modified version of the standard RECIST). As a result, the outcomes of this method met the assessment of specialists to an acceptable degree. However, the method is deemed a proof of principle supporting the feasibility of similar and more accurate tools. Along similar lines, other works addressing automated segmentation of lung cancer images are gathered in Table 8 with their reported evaluation metrics mentioned in Section 4.6. The cited research provides information on the class of deep neural networks used, the dimensional approach for lesion measurement, and the evaluation scores of the proposed methods. These DL approaches use 2D and 3D measurements rather than 1D measurements. Table 9 complements the information on the DL models presented in the previous table. It gives the size of the database and indicates how its use is distributed for training and for validation. Data on the architecture of the deep neural network models used are also given.

**Table 8:** Research addressing segmentation of lung cancer in CT images. Methods and score details.

Reference	Model Architecture	Dimensional Approach	Evaluation Score
[82]	CNN (U-Net)	3D	86.6% (ACC)
[83]	Mask R-CNN	2D	79.65% (ACC)
[79]	CNN (U-Net)	2D	72%(ACC), 75%(DC), 82%(SEN)
[84]	CNN (U-Net)	3D	82.8%(DC)
[61]	Mask R-CNN	2D	89.96%(ACC), 76.81%(DC), 87.72%(SEN), 86.7%(SPE)
[85]	CNN (MSDS-UNet )	3D	69.1%(DC), 74.4%(SEN)
[86]	GANs	2D	98.5%(ACC)
[87]	CNN (U-net)	3D	78% (DC)
[88]	CNN (SquExUNet)	3D	80%(DC)
[89]	CNN (SegNet)	2D	92.5%(ACC), 95.11%(DC), 98.33%(SEN), 86.67%(SPE)
[64]	CNN (ResNet50,U-Net)	2D	98.43%(ACC), 98.86%(DC), 98.99%(SEN)
[90]	CNN (U-Net)	3D	82%(DC)
[91]	CNN (GUNET3++)	2D	96% (DC)
[92]	CNN (SegChaNet)	3D	98.48% (DC)
[93]	CNN (RRc-Unet)	3D	87.77% (DC)
[94]	CNN (Unet)	2D	82%(ACC), 62%(DC)
[95]	CNN (RAD-UNet)	2D	88.13%(DC), 92.17%(SEN), 94.75%(SPE)
[67]	CNN and Transformer	2D	92%(DC)

ACC: Accuracy. DC: Dice Coefficient. SEN: Sensitivity. SPE: Specificity.

**Table 9:** Research addressing lung cancer in CT images. Data and DL method details.

Reference	Dataset	Image Resolution	Convolutional Layers	Deconvolutional and Max Pooling Layers
[82]	978 training	512 × 512	4 conv 3 × 3 × 32	3 maxpool
	419 validation		4 conv 3 × 3 × 80	3 deconv
			4 conv 3 × 3 × 160	
			2 conv 3 × 3 × 320	
			1 conv 3 × 3 × 1	
[83]	24,000 training	512 × 512	–	—
	8000 validation			
[84]	3000 training	512 × 512	2 conv 3 × 3 × 32	3 maxpool
			2 conv 3 × 3 × 64	3 deconv
			2 conv 3 × 3 × 128	
			2 conv 3 × 3 × 512	
			1 conv 3 × 3 × 1	
[79]	57,793 training	256 × 256 and	–	–
		160 × 160		
[61]	1265 training	512 × 512	–	–
[85]	15,553 training	512 × 512	1 conv 3 × 3 × 64	4 maxpool
	3196 validation		9 conv 3 × 3 × 1	4 deconv
			4 conv 3 × 3 × 2	
			1 conv 1 × 1 × 1	
[86]	23,400 training	512 × 512	–	–
	5200 validation			
[87]	3072 training	96 × 96	10 conv 3 × 3	4 maxpool
	768 validation		4 conv 3 × 3 × 2	4 deconv
			1 conv 1 × 1 × 1	
[88]	820 training	512 × 512		
	180 validation			
[89]	7400 training	–	28 conv	5 maxpool
	2600 validation			5 deconv
[91]	32,606 training	512 × 512	–	4 maxpool
				10 deconv
[92]	35,688 training	128 × 128	–	3 maxpool
	1750 validation			3 deconv
[93]	395 training	256 × 256	–	4 maxpool
	98 validation			4 deconv
[94]	12,000 training	512 × 512	–	–
	3000 validation			
[95]	7725 training	512 × 512	2 conv 3 × 3 × 64	4 maxpool
	1931 validation		2 conv 3 × 3 × 128	4 deconv
			2 conv 3 × 3 × 256	
			2 conv 3 × 3 × 512	
			2 conv 3 × 3 × 1024	
[67]	563 training	512 × 512	–	–
	113 validation			

The CT image databases used in research papers recapitulated in this section are shown in Table 10.

When discussing the time required to process CT images in cancer treatment monitoring systems, it is important to differentiate between the development phase of the deep learning (DL) model and the application of the resulting decision support system by radiologists. The design phase of the system involves training of the DL model, which consumes the majority of computational time. Therefore, our emphasis on computational efficiency mainly pertains to this design phase.

The training processes of DL models and the processing of CT images by these trained models consume varying amounts of computational time, influenced by several factors. This diversity of determinants makes it hard to compare approaches beyond the metrics reported. In this context, we present some experiments to give an idea of the computational time required for specific tasks and give insights into the factors influencing these times. One primary factor is the number of images being processed, along with any necessary pretreatment or normalization steps for those images. Additionally, the computational efficiency is affected by the hyperparameters of the model and can vary depending on the hardware specifications. Training a DL model can take hours, while making predictions with a trained model typically occurs in milliseconds or a few minutes per image. By way of example, we conducted classification experiments using a first set of 613 brain CT images with a resolution of 394 × 394 pixels, and a second set of 800 lung CT images with a resolution of 512 × 512 pixels. The training of a U-Net with an architecture designed by Sensio in 2021 lasted 5 h for the first set and 12 h for the second set, but classification predictions were generated in just seconds.

Moreover, to evaluate hardware performance, we conducted another experiment to train and test a deep neural network-based model for classification. We refer to a study by Alzamorra (2023) that introduced a DL approach for brain segmentation in magnetic resonance (MR) images. This method utilized a UNET model we trained with 325 MR images, while the other 325 segmented images served as ground truth. The authors of that study provided all the images. In our experiment, we trained the DL model for cancer prediction on two different hardware platforms. The first system was a workstation equipped with an Intel Xeon processor, 64 cores, and a clock speed of 3.0 GHz. The second system featured an Intel 5i processor with two cores and a clock speed of 2.6 GHz. The first setup required 1 h and 21 min for training, resulting in prediction times of 3.3 s. In contrast, training on the second system took 2 h and 48 min, with prediction times extending to 4.7 s. Notably, the second configuration, with lower performance specifications, took nearly twice as long to train and had prediction times that were 1.5 times longer than those of the first setup. As the number of processed images increases, the disparity in computational efficiency becomes more pronounced, especially during the training phase. Both the training and the prediction time can easily increase depending on the applied image preprocessing, the components of the DL method, and the level of automation achieved.

For clinical evaluations using the RECIST criteria, experienced radiologists point out that many factors influence the time spent to evaluate medical images. The process typically begins with analyzing and defining target lesions, which can take approximately 15 to 20 min for an experienced radiologist. Following this, measuring a CT scan can take an additional 10 to 15 min. Altogether, the estimated evaluation time can range from 35 to 40 min. However, this is not a standard time frame, as actual clinical cases can vary widely, potentially demanding more or less time.

The time required for image assessment using DL methods varies based on the specific DL technology used and the level of automation achieved, among other factors. Nevertheless, these methods perform competitively when compared with clinical professionals. For a comprehensive comparison of DL methods and healthcare professionals in disease detection from medical imaging, the work of Liu et al. (2019) [96] is recommended. The study examined various conditions such as ophthalmology, breast and lung cancer, dermatology, among others. Most studies utilized retrospective datasets, with 87.8% relying on retrospectively collected data and only 12.2% using prospective data. Regarding the DL models analyzed, several architectures were employed, with CNNs being particularly prominent in these studies.

In terms of model validation, internal validation was preferred over out-of-sample validation in 70% of the cases analyzed. In a comparison of 14 externally validated studies, the sensitivity scores were 87% for DL methods versus 86.4% for professional clinicians. The specificity scores were 92.5% for DL methods compared with 90.5% for clinicians. However, among 69 quantifiable studies out of 82 considered, the sensitivity and specificity scores decreased from 87% and 92.5% to mean values of 79.1% and 88.3%, respectively, for DL methods.

The results demonstrate comparable performance between DL models and healthcare professionals across various specialties. However, several limitations exist, including a lack of direct comparisons, few cases of external validation, and variability in the quality of study design and reporting. Furthermore, internal validation tends to overestimate the diagnostic accuracy for both DL and clinicians.

Recommendations for future application of DL methods should include the need for broader and standardized validation in clinical settings to prevent overly favorable results from internal testing. It is also important to improve transparency and methodological rigor, which currently hinder reproducibility and interpretation. To improve reporting quality, international reporting standards are necessary to facilitate the integration of DL in healthcare.

For the time being, DL should be considered an aid for healthcare applications rather than a replacement for clinicians. Finally, efforts are needed to streamline case triage and flag abnormalities effectively.

### 5.2. Underscoring the Role of Deep Learning in Measuring Metabolic Changes

Positron emission tomography (PET) is a standard imaging technique used for diagnosing lung cancer and evaluating treatments in efforts led by oncology physicians aiming to find a cure for this disease. PET imaging uses fluorodeoxyglucose (FDG) to characterize lesions within a physiological and metabolical frame of reference. This medium exposes the heterogeneous texture and variable contours of the nodules, revealing information that may not be apparent in CT images [97,98,99]. In contrast, metabolic and anatomical scans can provide complementary information. Similar to CT-based tests, metabolic evaluation using PET is a systematic method that requires a high degree of experience and consistent criteria to perform effectively. By its nature, the PET technique is prognostic, making it more effective and faster for evaluating treatment responses in solid cancers compared with anatomical assessments. However, various factors, including specific conditions for its use and limited availability of the necessary equipment in oncology centers, result in the less frequent use of PET in clinical evaluations. DL methods have been proposed in the literature that utilize PET images or combined PET/CT scans as the inputs for specially designed algorithms [65].

The following studies in the field are outlined to identify the engaged DL tools and objects, the image processing steps applied to PET images, and the various tasks involved in DL method designs that are practical for lung cancer detection and treatment monitoring, with a particular focus on the latter goal.

Recently, several deep learning methods have been developed for NSCLC detection. Zhang et al. [100] introduced a multiscale region-based CNN method that uses PET images to detect lung tumor candidates. This strategy uses three models of Mask R-CNN, which are functions are designed for object detection and segmentation. The models were tuned and trained with datasets at three different scales. A weighted voting or decision-making approach was implemented to reduce false-positive outcomes. The applied Max R-CNN is an extension of the R-CNN, also known as Region-based CNN, a simple and scalable object detection algorithm. An intermediate improved algorithm, Faster R-CNN, offers flexibility and robustness. It classifies the objects but does not find which pixels are part of an object in an image. Mask R-CNN enhances the Faster R-CNN by inserting a function to predict segmentation masks on the RoIs alongside the current classification and bounding box regression elements [101]. Its functions are object detection and semantic segmentation. Similarly, for NSCLC detection, Chen et al. [97] proposed a multimodal attention-guided 3D CNN method for analyzing combined PET/CT images. Attention mechanisms, which are a class of neural network layers allow the model to emphasize specific parts of the input by weighting inputs according to the relevance to the task at hand. As a result, this work registered an advantage in detecting cases that might otherwise go unnoticed through visual assessment.

Some studies have delved deeper into using DL for evaluating metabolic changes. Accurate tumor assessment in PET images requires effective segmentation of FDG uptake. A study directed by Früh et al. [68] aimed to develop a DL method for weakly supervised segmentation of tumor lesions from preprocessed PET/CT images. This approach simplifies labeling, thereby reducing the need for extensive human involvement during model training. The segmentation mask was designed in the following way. A radiologist manually annotated the lesion slice-by-slice, labeling them as “tumor” and “not tumor.” A CNN based on the VGG-16, architecture, comprising 16 layers with 13 convolutional and three fully connected layers, was then trained for classification. This architecture is recognized for its effectiveness in computer vision tasks such as image classification and object recognition. The proposed algorithm differentiates slices with and without FDG-avid tumor lesions. Subsequently, class activation maps (CAMs) or saliency maps were generated using several methods (CAM, GradCAM, GradCAM++, and ScoreCAM) to identify the tumor regions relevant to the network’s decision. Adaptive image segmentation was achieved in the region proposed by the CAM algorithm. The 3D Dice score, metabolic tumor volume (MTV), and total lesion glycolysis (TLG) metrics were used to estimate its performance.

The development of DL methods is confronted with the need to handle a large volume of images. To overcome this issue, Protonotarios et al. [102] presented a DL method utilizing a few-shot learning scheme based on the widely used U-Net architecture. The advantage of this approach lies in its ability to use less data for training. Furthermore, the strategy incorporates user feedback to continually improve accuracy in lung cancer lesion segmentation of PET/CT scans. For further advancements in DL methods, readers are referred to various works that develop techniques based on modified U-NET or V-NET as CNN architectures as classes of CNNs for image segmentation. These studies are regrouped in Table 11 along with their reported evaluation metrics mentioned in Section 4.6. The measurement approach is usually designed for 2D and 3D methods. The modified CNNs achieve a more precise and automated segmentation of the ROI in FDG-PET images. For the same studies, Table 12 provides details on dataset size and image resolution. The architecture of the DL models is likewise included. The Dice Coefficient, measuring similarity, has been the preferred metric for the DL methods based on PET scans, yielding values between 78% and 93%. In contrast, DL methods based on CT scans show a broader range of Dice Coefficient values from 69.1% to 98.86%. For the latter, Accuracy is more frequently reported, with rates from 72% to 98.5% in the reviewed studies.

The clinical evaluation of lung cancer treatments consists of comparing pre- and post-treatment measurements to determine the response according to criteria specified in Table 3, Section 3.1. To our knowledge, current research on the subject does not treat full automation for these measurements. The literature addressing DL methods relying on PET image processing presents various approaches to classify a positive or negative prediction of cancer treatments operating radiomic features extracted by CNN. In this regard, Amyar et al. [110] proposed a multiscale DL framework to predict survival and response to treatment of patients with esophageal and lung cancer. Their method aims to train a neural network, classify the pathology, segment the lesion, reconstruct the image, and predict the segmentation results. The U-Net was chosen as the backbone for processing 3D medical images. The main idea was to extract information from intratumoral and peritumoral regions to heighten classical radiomic assessments. Their strategy was to propose a multitask learning network to feed global features to the CNN in order to achieve tumor segmentation on 3D PET scans while driving the tasks of classification and reconstruction. The architecture comprised encoding and decoding parts (these last for reconstruction and segmentation) and skip connections between them. A multilayer perceptron (MLP) was used to classify a CNN for result prediction following the segmentation outcome.

A new strategy was proposed by Li et al. [66] by developing a DL model capable of accurately predicting PD-L1 (Programmed cell death protein-1) expression in NSCLC patients undergoing immunotherapy based on CT/PET data. This method provides a noninvasive tool for physicians to identify PD-L1-positive patients. In this approach, specialists outlined the ROI on medical images. This ROI, along with the original CT and PET images, was input into the PyRadiomic module, which is an open-source package written in Python (version 3.5) for extracting radiomic features from medical images. The cited work involved further feature extraction using high-dimensional image analysis techniques that employed image filters, such as Wavelet and Gaussian filters. A CNN with ResNet-101 architecture was used for feature extraction from PET/CT images. The ResNet-101 is a CNN that is 101 layers deep, and a pretrained version exists in the ImageNet database. A radiologist selected a rectangular bounding region enclosing the tumor. The information was manipulated to enter the feature extraction software. A logistic regression analysis favored the combination of radiomics and deep learning signatures to calculate the total probability of positive PD-L1. In the end, statistical methods helped to differentiate clinical features in positive and negative PD-L1 NSCLC patients. Furthermore, the model’s performance was statistically evaluated to determine its Accuracy, Sensitivity, and Specificity.

Some training databases were reported to be used in the cited research to collect PET images. Table 13 exhibits some of these available databases.

In terms of measurement in CT scans, pretreatment techniques can improve the quality of PET images. In turn, some processes, such as AI denoising methods, may slightly modify the information in the image in an unknown way [111]. Some studies have verified the influence of denoising methods, which include the works in Weyts et al. [112] and Jaudet et al. [111]. A relevant example is the study of Weyts et al. [65], who offered and attempt to investigate whether AI denoising for PET images has a measurable impact on lesion quantification during cancer treatment evaluations, in accordance with EORTC and PERCIST guidelines. These guidelines are known to yield very similar classifications. The study concluded that Images generated through AI-denoised PET images can be included and that treatment responses determined clinically are satisfactory according to the established EORTC and PERCIST criteria.

## 6. Practical Issues for Clinical Deployment of AI Technology

### 6.1. Regulation and Legislation Initiatives for AI Devices in Radiology

The increasing adoption of AI technologies in healthcare has sparked concerns about the potential replacement of radiologists with intelligent systems. However, it is unlikely that AI will completely take over the roles of radiologists in routine clinical practice anytime soon. Nevertheless, we can expect the integration of AI technologies into healthcare workflows in the near future. These advancements are anticipated to reduce discrepancies in care delivery and enhance precision in medical practices. This manuscript deals with AI technologies related to medical devices. It is important to clarify that not all healthcare AI software qualifies as medical devices. For instance, software that analyzes large datasets to generate insights about a particular disease may be primarily research-oriented and aims to advance medical knowledge. In contrast, medical devices have a direct impact on healthcare practices by providing, for example, treatment guidance for individual patients, which is the specific subject of this review. The term “medical device” refers to any instrument or tool, along with its accompanying software, designed for human use to prevent, diagnose, monitor, treat, or cure diseases [113]. One significant challenge in introducing these intelligent medical devices in healthcare institutions is ensuring their effective regulation.

This section delves into regulatory actions and legislation related to medical devices that integrate AI within the healthcare sector. Insights from Pesapane et al. [113] inform the regulatory framework for medical devices and data protection in the European Union (EU) and the United States (US). Based on that information, the first part of this section provides an overview of the legislative landscape in these regions.

In 2017, the European Parliament and Council adopted Regulation (EU) 2017/745, which governs medical devices. This regulation introduces stricter standards and obligations, updating earlier directives such as 90/385/EEC and 93/42/EEC. The Medical Devices Regulation (MDR) specifies requirements for medical devices, including the installation of quality management systems and protocols for post-marketing surveillance. Additionally, the EU places a strong emphasis on data protection through the General Data Protection Regulation (GDPR), which provides a framework for safeguarding personal data, including health-related information. Furthermore, the Cybersecurity Directive (2018), implemented by EU member states, outlines actions to prevent cyberattacks and protect essential services. In sum, the EU aims to ensure the safety, efficacy, and compliance of advanced legal frameworks for medical devices.

In the US, lawmaking developments highlight the need to balance technological advancements with data protection, privacy, and standards. The 21st Century Cures Act is one initiative that emphasizes the importance of data usage and privacy, particularly concerning AI technologies. This legislation aims to transform healthcare through technology by enabling precision medicine while prohibiting data marketing practices. Additionally, the Health Insurance Portability and Accountability Act (HIPAA) governs health information technology and mandates policies and training for individuals who access sensitive data. The US Food and Drug Administration (FDA) plays a crucial role in protecting public health by regulating medical devices to ensure their safety and efficacy. However, the regulatory process can be complicated due to the rapid pace of technological advancements. Unlike the EU, which has adopted strong cybersecurity directives such as the Cybersecurity Directive and GDPR, the US lacks comprehensive regulations to protect against cyberattacks and manage privacy issues. In summary, while the US approach requires more precise data handling policies and cybersecurity measures, it currently trails behind the EU in terms of the regulatory strength governing medical devices and digital security.

Regulation and legislation surrounding AI technologies are continuously evolving, with numerous stakeholders in the process. This groundbreaking structure aims to stimulate innovation while safeguarding user rights. Van Kolfschooten and Van Oirschot [114] gave an overview of the latest legal framework for AI systems. The EU’s Artificial Intelligence Act is set to impact various sectors, particularly healthcare. This legislation governs the development, market placement, deployment, and utilization of AI systems throughout the EU. The Act will influence national policies and practices as AI adoption accelerates in medical domains like diagnosis, treatment planning, and patient care. It establishes legal obligations for stakeholders, including technology developers, healthcare professionals, and public health authorities.

This landmark legal framework stimulates innovation within the healthcare sector that necessitates specialized implementation guidance. In this context, a regulatory "sandbox" serves a tool that enables the exploration of new products under supervision [115]. Recitals in the AI Act are introductory statements that provide the context for the legislation framework. One of such recitals in the AI regulatory sandbox, Recital 47, states that personal data collected legally can be used only for developing, training, and testing AI systems. This is particularly relevant for AI systems designed for public health, including disease detection, diagnosis prevention, control, and treatment, and improvement of health care systems [114,116,117].

The AI Act uses a risk-based approach to AI development, where the level of risk determines the strictness of regulations. The risks of AI systems are categorized as unacceptable, high, low, or minimal. These classes are defined for various AI products designed for specific tasks. For instance, AI-based tools for evaluating disease treatment may fall into the moderate to high-risk categories, as they are intended to support clinical decision-making [116,117]. In the health sector, gradually more sophisticated diagnostics and decision support systems are planned to be incorporated, which must be reliable and accurate.

Pesapane et al. [118] discussed the integration of AI into medical imaging, with a focus on mammography. The study highlighted the challenges of transforming research into a medical device while ensuring ethical and regulatory compliance under the EU MDR and the new EU AI Act. Key ethical considerations include transparency, patient privacy, and equitable access to healthcare. Pesapane et al. [118] outlined the process of collaboration between healthcare institutions and industry partners. An important feature of this collaboration is the need to ensure that AI models are robust and the resulting devices are clinically relevant while meeting strict compliance requirements. Overall, the study underscores the need for a strategic framework that balances innovation with ethical standards to improve both patient care and operational efficiency.

### 6.2. Validation Protocols

A study launched by the Panel for the Future of Science and Technology recommended a multitest approach to validate AI systems in healthcare, particularly in the imaging field [119]:

The first step is the feasibility testing, which evaluates algorithms under ideal and controlled conditions. This phase involves comparing the performance of AI systems with that of medical experts and other algorithms. Even if full robustness is not demonstrated, the results can still be subject to scientific publications. Next is the capability phase, where the AI system is tested by simulation or clinical trials in more realistic conditions. This phase is known as “in silico validation.” In this phase, the driven tests minimize the risk of harm from unexpected issues and require the participation of clinicians and operators. The outcome is a more reliable and improved AI system. The effectiveness phase shifts the testing to the clinical settings. The goal is to assess and optimize the performance of the AI system in real-world scenarios, beyond simulations. Any quality issues that arise during this phase are collaboratively addressed by developers and clinicians. This phase leads to an optimized design of the AI system. Finally, the durability phase involves performance monitoring and auditing mechanisms. This stage aims to continually enhance the deployment of the AI system by detection, notification, and correction of any issues that arise. At this stage, updates to the AI system can be implemented by training the algorithm with a larger dataset. This process is iterative, allowing for new tests to be conducted under controlled conditions before returning to clinical monitoring and installation. Most of the research collected in this review pertains to the feasibility step that promotes the progress and applicability of the DL technology.

### 6.3. Challenges and Future Directions, Enabling the Introduction of DL Methods into Current Clinical Practices and Workflows

Miotto et al. [120] discussed the challenges and future directions for DL methods in the healthcare sector. Meanwhile, Sousa Santos and Amorim-Lopes [121] examined the strengths, limitations, and areas for further research. A primary recommendation is that for DL models to be effectively integrated into existing clinical practices and workflows, developers of DL technology must collaborate closely with healthcare professionals and policymakers. This partnership requires training for both parties: healthcare professionals and DL-device developers.

In response to their research question concerning the validation of AI algorithms that assist patient-related decision-making in oncology practice, Sousa Santos and Amorim-Lopes [121] concluded after a thorough review of AI technology in medical applications that there is definite potential to revolutionize medicine and enhance healthcare quality. While the primary focus in the literature has been detection and diagnosis, the ultimate goal of AI technology is to have a broader impact on oncological patient care, which can include treatment assessment, the target area of our review. Several key challenges must be addressed when incorporating DL methods into clinical workflows. Specifically, when assessing lung cancer treatment responses, we identify the following correlated issues:

First, there is the case of data volume and quality. The successful training of DL models requires large healthcare datasets and a substantial amount of labeled data. Additionally, medical images often contain noise and irregular features, which complicate tasks such as object detection, feature extraction, and segmentation. The issue is to successfully develop systems based on larger sample sizes and standardized data.

Interpretability of results is a major concern in the use of DL models. These models are frequently seen as “black boxes”, making it difficult to comprehend how they arrive at their decisions due to their complex internal structures. This lack of clarity can undermine the trust of clinicians and limit the acceptance of DL medical devices in clinical environments.

Likewise, the rapidly advancing nature of DL techniques also requires continuous updates to model architectures and incorporation of new DL approaches.

From the perspective of developers, regulatory awareness is critical for successfully integrating DL into medical practice. Developers must stay informed about the evolving regulatory and legal frameworks surrounding AI technology. Furthermore, it is essential to ensure comprehensive validation of DL technologies, address regulatory considerations, and effectively integrate these innovations into existing clinical workflows.

The following trends and future directions for integrating DL methods into clinical workflows are currently being considered in response to the identified challenges:

To address dataset issues, feature enrichment involves leveraging diverse data sources. Examples include electronic health records (EHRs), social media, wearables, genomics, and environmental information, which aid in creating more comprehensive patient profiles.

Regarding DL models, the development course is towards enhancing trust in clinicians, attaining more impact on patient care, and impacting clinical research. Thus, developing interpretable modeling techniques is essential to explain how models arrive at their predictions, thereby fostering clinician trust in clinical decision-making aids. Then, the so-called temporal modeling pertains to designing architectures capable of handling time-dependent data to track disease progression and enhance disease classification and treatment assessment. Scalable Personalized Health Systems (SPHSs) engage multimodal data in applications like disease risk prediction and treatment recommendation. Moreover, Hypothesis-driven discovery is an approach that utilizes DL models to support exploratory analysis and hypothesis formulation in clinical research.

Federated inference is a concept for partnership work that entails developing models with the participation of multiple institutions. It aims to share raw data while preserving privacy and improving model generalization.

Ultimately, model privacy and security mechanisms should protect sensitive medical data while incorporating expert knowledge into models to improve their robustness and clinical relevance.

## 7. Research on AI Technologies in the Healthcare Sector

Comprehensive reviews have demonstrated significant advancements in artificial intelligence (AL), machine learning (ML), and deep learning (DL) within the context of cancer diseases. Table 14, Table 15, Table 16, Table 17, Table 18 and Table 19 summarize and highlight the key findings in the field. The main clinical targets identified in the literature compiled by reviews in the realm of DL methods for healthcare relate to the following:

1. Screening, which involves testing to detect cancer in individuals who do not exhibit symptoms.
2. Detection, which refers to identifying cancerous cells, often through imaging techniques or biomarker tests.
3. Diagnosis, which confirms the presence of the cancer, and defines the specific type, its stage, and other critical factors to formulate a treatment plan.
4. Other purposes encompass treatment assistance, such as planning and evaluating the response to treatment using standardized criteria. Prognosis and predictions, such as a mutation, are also contemplated.

Table 14 features general surveys focused on clinical support systems that assist with decision-making processes in healthcare. The primary topics covered include general methodologies from AI, ML, and DL technologies, alongside various medical objectives. Some papers also discuss issues related to the evaluation and implementation of these systems. Notably, the review by Elhaddad and Hamam [122] examines disease treatment recommendations that align with our area of interest.

The comprehensive reviews summarized in Table 15, Table 16 and Table 17 provide an overview of the general methods and procedures for image processing in the context of advancements in AI and ML. Key learning methods and DL models are examined in detail. Various research papers describe different types of learning, DL model architectures, available databases, and recent advancements in the field. The described methods cater to a range of tasks, including image reconstruction and generation to produce realistic synthetic images, as well as data augmentation for training sets. Essential processes for medical imaging encompass image pretreatment, preprocessing, postprocessing, and registration. Furthermore, these processes encompass object detection, computer-aided detection (CADe), computer-aided diagnosis (CADx), lesion segmentation and characterization, classification, and model training. Specific applications highlighted include data annotation approaches, linking image data to reports, and Content-Based Image Retrieval (CBIR). This technique enables image searches in databases based on visual content rather than metadata, facilitating the identification of similar cases, the recognition of rare disorders, and the enhancement of patient care. The medical applications of these methods involve diagnosis, evaluating malignancy to gauge disease severity, identifying mutations, and predicting survival outcomes. The studies presented in Table 15 direct attention to various types of cancers and other diseases. Furthermore, the research documented in Table 16 examines trends and future lines in DL models and methods. Additionally, the reviews in Table 17 introduce topics related to challenges, standards, and legislation within the field. In particular, the work by Sahiner et al. [129] emphasizes the assessment of treatment response.

**Table 16:** Reviews providing concepts and fundamentals on AI, ML, and DL technologies. Methods and models with a focus on medical image analysis. Trends and future directions.

Review Content	Authors
The study introduces ML problems and types. It outlines research on DL architectures and processing medical images, including image registration, object localization, classification algorithms, detection, training methods for classification, detection, and segmentation, clustering, dimensionality reduction, and Q-Learning. It settles trends, challenges and future directions in the field.	Suganyadevi et al. [130]
The paper introduces concepts and basic methods of AI, ML, and DL. It describes the learning types and the AI-based medical imaging analysis workflow. The paper gathers research on feature selection and extraction, models for regression and classification, and their main architectures. It states trends and future lines of research in the field.	Barragán-Montero et al. [131]
The reviewed image processing techniques and their advances include classification, object detection, segmentation, image generation (using GAN to create realistic synthetic images) to increase classification or segmentation accuracy, or to enable anomaly detection. It discusses open questions and future directions in the field.	Kim et al. [132]
The paper introduces learning types and DL methods, as well as their issues. The applications are for image registration, anatomical/cell structures detection, tissue segmentation, DL for computer-aided detection (CADe), computer-aided disease diagnosis, and prognosis. It identifies issues and future directions in the field.	Shen et al. [133]

The AI methods outlined in the referenced literature play a crucial role in cancer care applications. Table 18 and Table 19 collect a comprehensive overview of intelligent systems that can be integrated into the lung cancer care workflow. The literature included in these tables focuses on the screening, detection, and diagnosis of the disease. Additionally, other practices associated with the cancer care workflow must adhere to established medical standards and procedures. Our review is distinctive because it offers an in-depth analysis of standardized guidelines for assessing oncological treatments based on CT and PET scans for lung cancer. We incorporate research on advancements that are guided by these criteria.

Table 18 presents systematic literature reviews that answer specific research questions, primarily related to the detection and diagnosis of lung cancer. These questions involve recognizing key DL models, algorithms, and methods, as well as assessing their performance efficiency and relevant metrics. Additionally, due to the type of review, these analyze the sources, their origin, the quality of information, and statistics related to the types of studies reported. Future research directions are sometimes discussed as well. Table 18 presents narrative reviews, which constitute the majority of studies cited in the tables above as well as in the current review. These surveys provide an exhaustive synthesis of selected topics, covering a broader scope, but they do not employ a formal methodology for study selection. The reviews cited in Table 19 highlight specific and detailed advancements in DL methods for lung cancer screening and diagnosis.

**Table 17:** Reviews providing concepts and fundamentals on AI, ML, and DL technologies. Methods and models with a focus on medical image analysis. Challenges, standards, and regulations in the field.

Review Content	Authors
The survey outlines DL techniques, models, platforms, and resources for image processing and assessment. It explains research on transfer learning and fine-tuning of DL models, types of learning, and data augmentation for training sets. Also, data annotation through mining text reports and active learning. The tasks considered are image preprocessing, lesion segmentation, organ detection, tissue or lesion characterization, and model training. The medical applications involve: Diagnosis, prognosis and staging, measurement, treatment planning, and response assessment. The examined challenges to develop DL methods for medical image evaluation were linked to robustness (limited dataset size, availability of a large number of properly annotated cases, oversizing), repeatability, statistical estimation of performance, and interpretability.	Sahiner et al. [129]
The review describes advancements in DL models and their varied architectures. It outlines DL applications in healthcare and their optimization for segmentation and classification of images. It addresses topics such as data privacy, legal use, and standards.	Razzak et al. [134]

Our review comprehensively demonstrates the clinical relevance of NSCLC care and clinical treatments supported by medical imaging. We outline standard clinical criteria for assessing treatment responses in NSCLC, specifically using CT and PET imaging. Moreover, we present literature research by medical practitioners that cope with different dimensional measurement approaches and criteria for cancer treatment monitoring. The discussion then transitions to DL model architectures and other essential tools for medical image analysis. We concentrate on research related to automated and semi-automated methods for monitoring lung cancer treatment, detailing specific image processing tasks associated with these methods. Our emphasis also includes approaches based on measurements in one, two, and three dimensions as an extension of standard 1D measurements in CT scans. This capacity of handling large amount of images trough different measurement approaches is facilitated by the use of DL models.

To ensure completeness, we address challenges related to the practical implementation of these technologies in clinical settings. This content includes discussions on regulatory and legislative initiatives for IA developments in healthcare, validation protocols, as well as challenges and future directions for DL research within the healthcare sector.

**Table 18:** Reviews of DL techniques for screening, detection, and diagnosis of lung cancer.

Review Content	Authors
The research questions of the review address the following issues related to lung cancer detection by chest radiographs and CTs:	Nguyen [135]
RQ1: Publications over time. RQ2: Datasets for the training of DL models. RQ3: Training approaches for LD models. RQ4: Challenges in DL models for lung detection. RQ5: Future direction for DL lung cancer models.	
A comparison of studies for lung cancer detection was given, based on CT and CR, with existing surveys with respect to transferring data (feature extraction). The review considered CNN architectures for 2D and 2D-3D analyses. Comparison of DL models describing their architecture, and distinguishing between application (including mainly detection, diagnosis, early stage detection, classification, prediction of mutation ), database, pros and cons, performance metrics, mainly using ACC: Accuracy, AUC: Area Under the Curve, SPC: Specificity, SEN: Sensitivity or Recall, PPV: Precision or Positive Predictive Value, CPM: competition performance metric. Discussed databases and future directions.	
A systematic review methodology solves the research question on the relevance of lung cancer, with a focus on the diagnosis based on DL learning. It analyzes DL tools, classification algorithms, CNN architectures, datasets, evaluation criteria, and accuracy estimates of the performance for the resulting developments. Finally, it identifies universities active in the field.	Hosseini et al. [136]
The review provides a description and classification of lung cancer. It characterizes cancer therapies, cancer progression in stages, and analysis of lung cancer. The focus is on Imaging techniques for lung cancer detection. The work is presented as a systematic literature review with the following research concerns:	Javed et al. [137]
RQ1: Recognizing issues in lung cancer studies. RQ2: Recognizing solutions for cancer medical issues. RQ3: Valuing DL efficacy for solving lung cancer issues. RQ4: Recognizing publication channels for lung cancer research.	
An emphasis is placed on the literature sources. Their quality assessment was scored based on the proposed solution, contribution, future work, and results. Further information is related to imaging techniques, datasets, model architectures, feature extraction techniques, and image preprocessing methods. The survey identifies the DL methodologies for lung cancer detection and the evaluation performance criteria consistently used.	
A systematic review and meta-analysis with a research question concerning the lung cancer diagnostic performance of DL algorithms, based on specific criteria, such as pooled sensitivity and specificity.	Forte et al. [138]

**Table 19:** DL techniques for screening, detection, and diagnosis of lung cancer.

Review Content	Authors
The review describes imaging techniques for screening and diagnosing lung cancer, as well as DL-based techniques to aid these tasks. The research outlines applications, the DL approach, and its advantages and disadvantages. The focus is on classification and segmentation methodologies. A discussion is provided on gaps, challenges, limitations, and future directions.	Thanoon et al. [139]
The survey presents imaging techniques for lung cancer and pulmonary nodule detection. It studies DL-based techniques, DNN architectures, and performance metrics for DL algorithms, mainly with CNNs. The study outlines advances in image segmentation, nodule detection, and classification approaches for CT images. Il also discusses challenges and future research on DL techniques.	Wang [11]

## 8. Discussion

The potential for introducing AI in clinical settings is significant. Medical devices and clinical support systems can be designed to automate diagnostic processes, to aid therapeutic decision making, and to conduct clinical research. The current paper provides an in-depth review of current research on deep learning (DL) methods that have been developed within the field of artificial intelligence (AI) to support medical practice in treating non-small-cell lung cancer (NSCLC). To begin with, the standard criteria that medical specialists use to monitor an oncology patient’s response to treatment were outlined. The relevant guidelines for CT and PET scans, the two main diagnostic and follow-up imaging tests for cancer, are also documented.

The information compiled in this survey includes a description of deep neural network (DNN) architectures. It also gives insights into DL tools that facilitate various image processing tasks, as illustrated in the flow diagram described in Figure 3 of Section 3. A general DL method consists of multifaceted functions, such as image labeling, model training, and model handling through different computational tools, which are also considered in this review. Further methods that can be incorporated deal with image pretreatment and classification of target lesions. A significant number of works are referenced for the reader’s benefit. This paper also provides information about image databases and metrics for evaluating the performances of the proposed methods. Although diverse metrics can evaluate the performance, the rating is often based on the accuracy (%) and the Dice Coefficient calculation. Thus, integrating DL into clinical practice needs a systematic approach.

Segmentation is a crucial step in DL methods. The findings of most of the reviewed studies reveal that DL techniques achieve high accuracy for this purpose. One of the strengths of these techniques is their relatively short processing time for data. However, training the model is time- and computationally intensive. The lesion segmentation undertaking is often performed by accessing extensive labeled databases. NSCLC classification for diagnostic purposes has demonstrated good accuracy in identifying lesions in clinical images. These approaches emphasize the use of filters to improve disease detection. In contrast, these filters are seldom applied in segmentation-focused research. The accuracy of the DL methods for clinical applications depends on several factors. These include the quality and quantity of the images used as input data, the hardware performance, the specific level of automation of the designed system, and the existing expertise, among others. However, comparable performances have been demonstrated between DL models and healthcare professionals, as discussed at the end of Section 5.1.

Another area of research involves strategies to improve the performance of deep neural networks and the overall efficiency of the DL methods. Some issues addressed in the literature and discussed in this paper include the robustness of CNNs as feature extractors in visual matching applications under varying conditions, such as changes in appearance, scale, and perspective. Other studies were concerned with analyzing the effects of AI denoising methods, which, while improving image quality, may slightly alter the information derived from the images in unknown ways. These minor changes have been verified in the output of cancer lesion quantification and treatment evaluation methods, without significant impact on the results.

This survey is framed around DL methods for monitoring NSCLC treatments, especially from CT and PET scans. Lesions on CT images are currently measured using different approaches including 1D, 2D, and 3D lesion measurements. One-dimensional measurements on CT images follow the RECIST guidelines and procedures. However, numerous investigations concern DL methods with surface and volumetric assessments in CT images, while PET scans consistently utilize volumetric measurements. This review distinguishes between these approaches, examines their challenges and outcomes, and presents various computational resources and neural network architectures applied in developing DL-assisted monitoring methods using CT and PET scans. The degree of automation in these methods is also discussed. Often, inputs to DL methods involve manual annotations and ROI selection, leading to semi-automated strategies. A notable reference is provided by Liu et al. [140], who developed a highly automated system for cancer follow-up that includes 1D measurement from MRI scans and an algorithm to segment liver metastases following the RECIST 1.1 criteria and based on 3D U-net trained for segmentation. Liver and liver metastases annotations were made by means of the open-source software platform (ITK-SNAP). The DL algorithm calculated tumor size, and a rule-based program evaluated treatment response according to RECIST 1.1 criteria. The tasks proposed in this work can potentially be adopted to enhance medical practice in NSCLC detection and treatment. Emerging technologies related to AI are rapidly evolving, and substantial efforts are being made to establish a regulatory framework as detailed in Section 6. This review offers an overview of the current regulations and legislative initiatives regarding AI-based medical devices. According to the existing validation protocols for medical devices, most of the related research found in the literature is still in the feasibility stage. However, it is crucial for academic research focused on developing AI or DL devices to be integrated into this regulatory framework. This integration is essential for progressing through the subsequent phases of validation, which include assessing capability, effectiveness, and durability. The ultimate goal is to create products that positively impact and support medical practice.

## 9. Conclusions

This literature review focuses on advances and tools enhanced by Deep Learning (DL) techniques for monitoring NSCLC treatment monitoring and the efforts to achieve automated methods. The main advancements in this field lie in the segmentation methods, as it is a fundamental step in automating DL-based clinical processes. DL neural network architectures can accomplish this task faster than a specialist for both morphological and metabolic tests. However, a significant challenge remains in model training, due to its time consumption and overall effectiveness, which needs to be addressed. A more lightly considered approach is predicting the response from the radiomic features extracted from the images. This prediction differs from the classical classification methods, which are largely guided by reference standards for treatment evaluation methods. On the other hand, the two-dimensional measurements in CT mages, which serve as a primary step before segmentation by neural networks, are still manually attained. For PET-based methods, the analysis is regularly performed using volumetric measures. To date, no DL methods have been reported to evaluate NSCLC treatment through fully automated metabolic clinical evaluations.

Our findings indicate that regularized neural network types implemented in the various DL methods aid cancer detection and treatment monitoring. These methods are primarily based on CNN, VAE, and GAN structures, including hybrid methods that combine different structures. For segmentation tasks, the U-Net architecture is the predominant choice. Moreover, many works have enhanced the performance of DL models by slightly modifying the named structures. We have identified the potential for adding preprocessing AI tools into clinical images, as only a few reviewed studies on the subject of cancer treatment control use image-filtering techniques. However, research focused on diagnosis has shown that specific filters can improve model precision. In addition, it has been proven that AI-denoising modifies image information, but these changes do not negatively affect the effectiveness of treatment evaluation. The reviewed research also considers issues about introducing robustness of neural network features and adaptive online parameter weighting to improve the accuracy of the neural network models.

Improving the volume and quality of clinical data presents a significant challenge. This advancement necessitates feature enrichment to effectively utilize diverse and standardized data sources. A major issue is the lack of interpretability, which leaves deep learning (DL) users uncertain about how models arrive at their decisions. One way to address this problem is through temporal modeling, which enables the analysis of time-dependent data and facilitates the tracking of disease progression. Continuous updates to systems are also essential to enhance model training and improve accuracy. Furthermore, the successful implementation of AI systems requires federated inference, which involves collaboration between institutions, including developers and clinical practitioners. The introduction of AI technology should aim to create scalable personalized health systems that entail effective treatment management.

In 2024, the latest legal framework for AI systems, known as the EU’s Artificial Intelligence Act, was introduced. This act is based on a risk-based approach to AI development, evaluating risks based on the severity of potential harm, as well as the likelihood and frequency of such harm occurring. Treatment monitoring through DL methods falls into the moderate-to-high-risk category. To minimize risks associated with AI development, it is essential to identify, monitor, and analyze potential risks for every new AI application. The risk assessment process should involve both developers and clinicians, starting during the validation phase and continuing through the effectiveness and durability phases, which include implementation in clinical settings and external auditing mechanisms.

## Figures and Tables

**Table 1:** Oncology and radiology literature that copes with the efficacy of different dimensional measurement approaches and criteria in cancer treatment monitoring.

Main Object	Criteria	Measurement	Type of Cancer	Reference
Compare different measurement approaches.	RECIST	1D, 2D and 3D	Lung	[23]
Compare different criteria	WHO and RECIST	1D and 2D	Gastric, lung, breast and liver	[24]
Compare different slice intervals.	WHO and RECIST 1.1	1D, 2D and 3D	Lung	[25]
Compare measurement approaches (3D spherical and ellipsoid vs. 1D).	RECIST 1.1	1D and 3D	Lung	[26]
Examine correlations between 1D and 3D measurements.	RECIST 1.1	1D and 3D	Lung	[27]
Analyze RECIST with various tumor types and different treatments.	RECIST 1.1	1D	Lung, breast and colon	[28]

**Table 2:** RECIST criteria for evaluation of cancer treatments.

Type of Lesion	Response Criteria	Condition
Target lession	Complete response (CR)	Disappearance of all target lesions or any pathological lymph nodes.
	Partial response (PR)	At least a 30% decrease in the sum of the target lesion diameters.
	Progressive disease (PD)	At least a 20% increase in the sum of target lesion diameters and demonstrate an absolute increase of at least 5 mm. The appearance of one or more new lesions is also considered progression.
	Stable disease (SD)	No contraction or increase sufficient to qualify as a PR or SD.
Non-target lesion	Complete response (CR)	Disappearance of all non-target lesions.
	Progressive disease (PD)	Unequivocal progression of existing non-target lesions. The appearance of one or more new lesions is considered progression.
	Stable disease (SD)	Persistence of one or more non-target lesions.

**Table 3:** Evaluation of cancer lesions according to EORTC and PERCIST.

Response Criterion	EORTC	PERCIST
Complete metabolic response (CMR)	Complete resolution of FDG uptake in all lesions.	Complete resolution of FDG uptake in all lesions.
Partial metabolic response (PMR)	Greater than 25% reduction in the sum of SUVmax after more than one cycle of treatment.	A minimum of 30% reduction in the peak lean body mass SUV (SULpeak) and an absolute drop of 0.8 SULpeak units.
Progressive metabolic disease (PMD)	More than 25% increase in the sum of SUVmax or appearance of new FDG avid lesions.	More than 30% increase in the SULpeak of the FDG uptake and an absolute increase of 0.8 SULpeak, or appearance of FDG-avid new lesions.
Stable disease (SD)	Not qualify for CMR, PMR or PMD.	Not qualify for CMR, PMR or PMD.

**Table 4:** Summary of the DL Networks.

DL Network	Main Applications	Reference
CNN	Image processing (detection, modeling, segmentation)	[34]
RNN	Time-Series Analysis, Language Processing (Modeling, analysis, translation) and Speech Recognition	[35]
RvNN	Molecular structure analysis and language processing (statistical modeling, analysis)	[36]
DBN	Image recognition, speech recognition and natural language processing	[37]
DBM	Images recognition and speech recognition	[38]
GAN	Generate images, complete missing information and generate 3D models from 2D data	[39]
VAE	Generate new data	[40,41]

CNN: Convolutional Neural Network. RNN: Recurrent Neural Network. RvNN: Recursive Neural Network. DBN: Deep Belief Network. DBM: Deep Boltzmann Machines. GAN: Generative Adversarial Network. VAE: Variational Autoencoder.

**Table 5:** Activation functions: (a) Sigmoid, (b) Hyperbolic Tangent, (c) Rectified Linear Unit, (d) Exponential Linear Unit.

$\sigma (x)=\frac{1}{1+e^{-x}}$	$\tanh(x)=\frac{2}{1+e^{-2x}}-1$
(a)	(b)
ReLU(x)=max(0,x)	$\mathrm{ELU}(x)=\max(0,x)+\min\left(0,1\left(e^{x}-1\right)\right)$
(c)	(d)

**Table 6:** Confusion matrix for a binary segmentation task.

		Ground Truth	
		Functional Tissue Units (1)	Background (0)
Prediction	Functional Tissue Units (1)	TP	FP
	Background (0)	FN	TN

Note: TP: number of FTU pixels properly classified as FTU. FN: number of FTU pixels misclassified as background. FP: number of background pixels misclassified as FTUs due to misalignment. TN: number of background pixels properly classified as background.

**Table 7:** Filters used for image preprocessing in DL methods reviewed in Section 5.

Type of Filter	Name	Reference
Noise removal	Wiener Filter	[64]
	Gaussian Filter	[65]
	Wavelet Filter	[66]
Blurring	Gaussian Filter	[67]
Attenuation corrected	—	[68]

**Table 10:** Training database in articles based on TC images.

Category	Database Name	Reference
CT lung cancer scans with manual delineation	NSCLC-Radiomics: https://www.cancerimagingarchive.net/collection/nsclc-radiomics/ accessed on 10 January 2025	[78]
CT lung cancer scans treated with anti-PD1	Anti-PD-1Lung: https://www.cancerimagingarchive.net/collection/anti-pd-1_lung/ accessed on 10 January 2025	[79]
CT scans with labeled nodules	Lung Nodule Analysis 2016 (LUNA16): https://luna16.grand-challenge.org/Data/ accessed on 10 January 2025	[64,82,94]
CT scans with labeled (lung nodules, livers tumors and enlarged lymph nodes)	DeepLesion: https://nihcc.app.box.com/v/DeepLesion accessed on 10 January 2025	[76,77]
CT scans with labeled lung cancer	LIDC-IDRI: https://www.cancerimagingarchive.net/collection/lidc-idri/ accessed on 10 January 2025	[83,85,88,91,94,95]
CT scans with labeled nodules	QIN-LungCT-Seg: https://www.cancerimagingarchive.net/analysis-result/qin-lungct-seg/ accessed on 10 January 2025	[78,86]
CT scans with labeled nodules	RIDER Lung CT: https://www.cancerimagingarchive.net/collection/lidc-idri/ accessed on 10 January 2025	[78,92]

**Table 11:** Articles based on segmentation of ROI in PET images.

Reference	Model Architecture	Dimensional Approach	Evaluation
[103]	CNN (U-Net)	3D	93%(DC)
[104]	CNN (V-Net)	3D	83%(DC)
[105]	CNN (mU-Net)	3D	87%(DC)
[106]	CNN (U-Net)	3D	78%(DC)
[107]	CNN (R-CNN)	2D	84%(DC)
[108]	CNN	2D	86%(DC), 83%(SEN)
[109]	CNN (U-Net)	3D	84.4%(DC), 83%(SEN), 84%(SPE)

DC: Dice Coefficient. SEN: Sensitivity. SPE: Specificity.

**Table 12:** Research addressing lung cancer in PET images. Data and DL method details.

Reference	Dataset	Image Resolution	Convolutional Layers	Deconvolutional and Max Pooling Layers
	3D			
[102]	–	256 × 256	1 conv 1 × 1 × 1	3 maxpool
			5 Conv 3 × 3 × 16	3 deconv
			6 Conv 3 × 3 ×32	
			6 Conv 3 × 3 ×64	
			3 Conv 3 × 3 ×128	
[103]	50 training	–	–	–
	26 validation			
[104]	48 training	512 × 512	2 conv 3 × 3 ×16	2 maxpool
	36 validation		2 conv 3 × 3 × 64	2 deconv
			2 conv 3 × 3 × 256	
			1 conv 3 × 3 × 1	
[105]	–	–	1 conv 3 × 3 × 2	3 maxpool
			3 conv 3 × 3 × 16	3 deconv
			4 conv 3 × 3 × 32	
			6 conv 3 × 3 × 64	
[106]	730 training	512 × 512	10 conv	4 maxpool
	81 validation			4 deconv
[107]	63 validation	256 × 256	–	–
[108]	88 training	128 × 128	–	–
	47 validation			
[109]	–	144 × 144	1 conv 3 × 3 ×3	4 maxpool
			4 conv 1 × 1 × 1	4 deconv
			21 conv 3 × 3 × 3	

**Table 13:** Training database in articles based on PET images.

Category	Database Name	Reference
PET/CT scans labeled Lung Cancer Diagnosis	Lung-PET-CT-Dx: https://www.cancerimagingarchive.net/collection/lung-pet-ct-dx/ accessed on 10 January 2025	[102,107,108]
PET/CT pretrained	ImageNet	[66]
PET or PET/CT scans labeled	Private	[68,97,100,103,104,105,106,109,110]

**Table 14:** AI for clinical support system (CDSS) decision-making.

Survey Target	Review Content	Authors
AI technologies CDSSs	The study delves into ML algorithms, natural language processing (NLP), and DL theory and applications. The survey includes advancements for diagnosis, treatment recommendations, risk prediction, early intervention, and clinical documentation. It briefly discusses usability, ethical and legal implications, challenges, and opportunities.	Elhaddad and Hamam [122]
	The study presents advances in ML, particularly computer-aided detection (CADe), computer-aided diagnosis (CADx), and decision support systems.	Giger [123]
Evaluation of CDSSs	The primary topic concerns the challenges in evaluating AI-based CDSSs. It encompasses the design, development, use, and continuous surveillance stages.	Magrabi et al. [124]
CDSSs implementation issues	The study identifies clinician issues on “trust” and “trustworthiness” by developing the following main aspects: control in terms of norms of clinical practice, autonomy, medical errors, and legal responsibility. The analysis encompasses both the clinician and patient perspectives.	Jones et al. [125]

**Table 15:** Reviews providing concepts and fundamentals on AI, ML, and DL technologies. Methods for various diseases and clinical objectives.

Review Content	Authors
The review examines ML for medical imaging, including preprocessing, segmentation, and post-processing tasks. The review brings closer to DL methods and model architectures, mainly for detection and classification, used to estimate malignancy. Diseases investigated were skin, lung, brain, and breast cancer.	Tajidini [126]
The review begins with a description of the medical image characteristics, clinical requirements, and applications. It provides advancements in DL technology, including architectures, annotation approaches (transfer learning, domain adaptation, self-supervised learning, semi-supervised learning). Also, weakly/partially supervised and unsupervised learning, embedded knowledge into learning (enhanced learning from hybrid imaging techniques, for example), and federated learning (with robust algorithmic models). It also covers advances in image reconstruction, pretreatment, segmentation, registration, detection (CADe), diagnosis (CADx), and classification for diagnosis. Medical cases presented concern:	Zhou et al. [127]
Thoracic imaging, such as diagnostic in chest radiography, lung cancer screening, and COVID-19. Neuroimaging: tissue segmentation and classification, prediction, registration, and other neuroimaging synthesis tasks with GANs. Cardiovascular imaging for cardiac segmentation, cardiac vessel segmentation, and cardiac motion tracking. Abdominal imaging, anatomy localization and segmentation, screening. Microscopy imaging: nuclei detection and segmentation, disease grading, mutation identification, survival prediction,	
The study primarily concerns ML types and CNN models.	
The survey describes several aspects of DL-based tasks undertaken in medical applications using image processing:	Litjens et al. [128]
DL tools for image classification, object detection, segmentation, registration. Image generation and improvement, linking data images and reports, and Content-Based Image Retrieval (CBIR).	
The research gathered and described involves CNN, RNN, SAE, AE, DQN, DBN neural networks architectures. The referenced studies concern image processing of anatomical areas, including neuro, retinal, pulmonary, breast, cardiac, abdominal, and musculoskeletal, obtained from CT, MRI, X-ray, microscopy, ultrasound (US), or video cervigrams.	
